# Gut microbiota-regulated unconjugated bilirubin metabolism drives renal calcium oxalate crystal deposition

**DOI:** 10.1080/19490976.2025.2546158

**Published:** 2025-08-24

**Authors:** Shujue Li, Wenzheng Wu, Yuhao Zhou, Shike Zhang, Daqiang Wei, Mingzhao Zhu, Xiaoling Ying, Xinyuan Sun, Hongxing Liu, Wei Zhu, Daolin Tang, Renjie Jiao, Guohua Zeng, Xiaolu Duan, Jinbao Liu, Wenqi Wu

**Affiliations:** a Department of Urology, The Second Affiliated Hospital, Guangzhou Medical University, Guangzhou, China; b Department of Urology, The First Affiliated Hospital, Guangdong Provincial Key Laboratory of Urological Diseases, Guangdong Engineering Research Center of Urinary Minimally Invasive Surgery, Robot and Intelligent Equipment, Guangzhou Institute of Urology, Guangzhou Medical University, Guangzhou, China; c Department of Surgery, UT Southwestern Medical Center, Dallas, TX, USA; d Sino-French Hoffmann Institute, Guangzhou Medical University, Guangzhou, China; e Affiliated Cancer Hospital & Institute of Guangzhou Medical University, Guangzhou Municipal and Guangdong Provincial Key Laboratory of Protein Modification and Disease, State Key Laboratory of Respiratory Disease, School of Basic Medical Sciences, Guangzhou Medical University, Guangzhou, China

**Keywords:** CaOx crystal deposition, gut microbiota, unconjugated bilirubin, oxalate secretion

## Abstract

Gut microbial dysbiosis and the resultant metabolic disorder are intimately associated with calcium oxalate (CaOx) stone formation. Renal CaOx crystal deposition is one of the primary initiating factors of CaOx formation; however, the critical signaling metabolites communicating along the gut–kidney axis, and their regulation on renal CaOx crystal deposition remain unclear. Here, we investigate the role of gut microbiota-associated unconjugated bilirubin (UCB) metabolism in renal CaOx crystalline pathogenesis. The UCB was first distinguished as a significant risk factor of renal CaOx crystal deposition, by transplantation of fecal microbiota derived from healthy rat (healthy-FMT) to alleviate the renal CaOx crystal deposition in rat models, which was also testified in CaOx stone patients. Further experiments showed that UCB could increase renal CaOx crystal deposition significantly in both rat and Drosophila models. Mechanistically, UCB can promote apoptosis in renal tubular epithelial cells, enhance oxalate secretion by upregulating Slc26a6 expression, and facilitate CaOx crystal nucleation and aggregation, all of which contribute to renal CaOx crystalline pathogenesis. Furthermore, we identified significant gut microbiota dysbiosis in renal CaOx crystal deposition rats, particularly in β-glucuronidase (β-GD) and bilirubin reductase (BilR)-related dysbiosis, which modulate UCB levels and its enterohepatic circulation. These findings suggest that UCB is a novel regulator of renal CaOx crystal deposition, and targeting its metabolism via modulation of the gut microbiota may offer a promising therapeutic strategy for preventing renal CaOx crystal deposition-related nephropathy.

## Introduction

Kidney stones are associated with renal fibrosis, chronic kidney disease, and an increased risk of renal cancer, which contributes significantly to escalating health-care costs. The present focus of research has been on CaOx stone, given its prevalence. Renal CaOx crystallization is a disorder characterized by hyperoxaluria, increased renal cell injury and crystal-cell adhesion, which are the key to CaOx stone formation.^1^ Despite significant advances in understanding the pathogenesis of renal CaOx crystal deposition, the systemic mechanisms, particularly the roles of gut microbiota and metabolic regulators, remain incompletely understood.

Recent research has identified significant alterations in metabolic pathways in patients with CaOx stones, emphasizing the complex regulation of metabolites both from the host and the gut microbiota.^2–5^ Distinct differences in microbial composition have been observed between individuals with CaOx stones and those without.^6,7^ These microbial changes, particularly in the metabolism of short-chain fatty acids (SCFAs) and arginine are implicated in the pathogenesis of renal CaOx crystal deposition.^8,9^ Given that metabolites regulated by gut microbiota can enter systemic circulation and influence distant organs, further investigation into gut microbiota-regulated metabolites is essential to uncover novel pathways contributing to renal CaOx crystal deposition.

Bilirubin, a yellow pigment derived from heme metabolism,^10^ exists in two forms: conjugated bilirubin (CB) and unconjugated bilirubin (UCB). As a component of bile, CB is secreted into the gut, where it undergoes microbial biotransformation into UCB, and further reduction into stercobilin.^11^ While bile acids have been shown to regulate oxidative stress, inflammation, and lipid peroxidation of the kidney,^12–14^ and alterations in bile acid metabolism are considered potential biomarkers for rats with renal CaOx crystal deposition,^15^ the specific role of UCB and its microbial metabolism in renal CaOx crystal deposition has received limited attention.

In this study, we utilized multiple models, including rat models, *Drosophila*, and human samples, to demonstrate the critical regulatory role of gut microbiota-regulated UCB activity on renal CaOx crystallization. This findings not only provide novel insights into gut microbiota-regulated metabolic mechanisms underlying renal CaOx crystal deposition, but also establish a potential approach to inhibit CaOx nephropathy.

## Materials and methods

### Animal experimental design and sample collection

Eight-week-old male Sprague Dawley (SD) rats were obtained from the Guangdong Medical Laboratory Animal Center (Guangzhou, Guangdong, China) and subsequently fed freely with sterilized water and food in a specific pathogen-free room at the Animal Experimental Center of Guangzhou Medical University's Second Affiliated Hospital. All experimental procedures were performed in compliance with the guidelines of the National Institutes of Health Guide for the Care and Use of Laboratory Animals.

To explore the effect of gut, microbial reconstruction and the key gut microbiota-regulated metabolite on renal CaOx crystal deposition, 24 rats were randomly divided into Control group (fed with standard chow diet), Crystal deposition group (fed with chow diet containing 5% hydroxyproline, HYP), and Crystal deposition + FMT group (HYP-feeding + health fecal microbiota transplantation, healthy FMT). Healthy FMT was performed in the previous description.^16,17^ In brief, fresh fecal pellets of 8-week-old healthy male SD rats that were fed with sterilized water and a standard chow diet were collected, combined, and resuspended using pre-cooled sterile PBS (200 mg/mL), followed by centrifuged under sterile conditions (1000 rpm, 10 min, 4°C). Then, the healthy microbial suspension was mixed with an equal volume of 20% sterile glycerol solution, split and stored at −80°C. Before FMT, the healthy microbial suspension was rewarmed, and recipient rats were transplanted with 200 µL microbial suspension every 2 days using gavage. After 45 days of administration, fecal samples were collected and rapidly frozen in liquid nitrogen and stored at −80°C for metabolomics profiling analysis and metagenomic sequencing analysis. Rats were euthanized by intraperitoneal injection of 2% pentobarbital, in accordance with ethical guidelines.

To explore the circulatory UCB activity alteration of renal CaOx crystal deposition individuals, 10 rats were randomly divided into Control group (sterilized water-drinking) and Crystal deposition group (0.75% ethylene glycol, EG-drinking); to explore the regulatory effect of UCB on renal crystal deposition, 16 rats (0.75% EG-drinking) were randomly divided into Crystal deposition group (1% DMSO gavage once every other day, v/v) and Crystal deposition + UCB group (20 mg/kg UCB gavage once every other day). After 30 days of administration, urine samples of 24 hours were collected using metabolic cages, preparing for urinary oxalate detection; serum samples were collected using blood collection tubes containing an inert separation gel and a coagulation reagent followed by centrifugation, kidneys were collected for further crystal deposition detection and IHC detection. The study was approved by the Second Affiliated Hospital of Guangzhou Medical University Ethics Committee (Approval No. B2023–129).

Screening healthy human and fecal sample of healthy human were performed at the Department of Gastroenterology, Second Affiliated Hospital of Guangzhou Medical University. The study was approved by the Second Affiliated Hospital of Guangzhou Medical University Ethics Committee (Approval No. B2023-ks-35).

### Analysis of renal CaOx crystal deposition

For rat model, the renal tissues were fixed and paraffin-embedded, and subsequently sectioned into slices of 4 µm in thickness. After Hematoxylin & Eosin (HE) staining, the deposition of CaOx crystals in the kidneys was observed using a polarizing microscope (CX31 Olympus, Tokyo, Japan), and quantified by estimating the area and perimeter of the crystals using WZCamera software.

For *Drosophila* model, the flies were dissected in phosphate-buffered saline, followed by evaluation of renal crystal deposition in Malpighian tubules under a polarizing microscope. All four Malpighian tubules of each fly were included, and the total area of CaOx crystals in the whole field of view was quantified using WZCamera software. Given the distinct anatomical characteristics of Malpighian tubules between the sexes, the crystal areas of female and male flies were segregated for statistical analysis.

### Metabolomics profiling analysis

Metabolomics profiling analysis was performed using ultrahigh-performance liquid chromatography – mass spectrometry (UHPLC-MS/MS) with a Vanquish UHPLC system coupled to an Orbitrap Q ExactiveTM HF-X mass spectrometer. The data files generated by UHPLC-MS/MS were processed using the XCMS to peak alignment, peak picking, and quantitation for each metabolite. After that, peak intensities were normalized to the total spectral intensity by the first sample. Then, based on the set 10 ppm mass deviation and adduction, compare it with the high-quality secondary spectrum database for metabolite identification to obtain the accurate qualitative and relative quantitative results. Then, background ions are removed using blank samples. The raw quantification results are then standardized according to the formula: original quantification value of the sample/(total quantification value of metabolites in the sample/total quantification value of metabolites in the first sample), to obtain the relative peak area. Statistical analyses were performed using the statistical software R (R version *R*-3.4.3), Python (Python 2.7.6 version) and CentOS (CentOS release 6.6). When data were not normally distributed, standardize according to the formula: sample raw quantitation value/(The sum of sample metabolite quantitation value/The sum of first sample metabolite quantitation value) to obtain relative peak areas. For comparative analysis, metabolite abundances between two groups were calculated by T test, among three groups were computed by ANOVA, and False Discovery Rate (FDR)-corrected p-values were calculated by Benjamini and Hochberg multiple comparisons. The R language package “ropls” (v 1.4.6) was utilized to conduct Orthogonal Partial Least-Squares Discriminant Analysis (OPLS-DA). Volcano plots were generated using the R language package ‘‘ggplot2,’’ and the R language package ‘‘pheatmap’’ was used to generate clustering heatmaps.

### Urine and serum biochemistry of rats

The concentrations of oxalic ions in 24 hours urine samples of rats were determined by ion-exchange chromatography (Metrohm, Herisau, Switzerland), and the levels of urinary UCB, urobilinogen, and urobilin were extracted from the metabolomic results. The levels of creatinine and urea nitrogen were determined using atomic absorption spectrophotometry. Serum TBIL and DBIL were determined with diazo reagent method, followed by a calculation of UCB concentration.

### Experimental design of Drosophila study and sample detection

The Drosophila study was conducted using wild-type D. melanogaster (w1118 strain; Bloomington Drosophila Stock Center). The fruit flies were reared on a standard diet comprising agar, sugar, corn meal, yeast and antiseptics, maintained at 25°C with a 12 h–12 h light–dark cycle and 50% humidity. The simultaneously emerging fruit flies were maintained on a standard food source for a three-day aging period, followed by transferring to hyper-oxlate food (NaOx, 0.1%, w/v) or normal food. For the treatment of UCB, a stock solution of UCB was prepared in DMSO and was diluted to indicate concentration in food, and the food was refreshed every day to ensure the activity of UCB. Flies fed with hyper-oxlate food were randomly divided into Crystal deposition group (0.1% NaOx diet), Crystal deposition +0.2 mM UCB group (0.1% NaOx +0.2 mM UCB) and Crystal deposition +2 mM UCB group (0.1% NaOx +2 mM UCB), followed by dissecting after 7 days of administration. Flies fed with normal food were randomly divided into the Control group (normal diet) and UCB group (normal diet +2 mM UCB), followed by dissecting after 27 days of administration. The flies were dissected in phosphate-buffered saline, followed by evaluation of renal crystal deposition formation in Malpighian tubules under a polarizing microscope.

### Cell lines, cell culture, and treatment

Canine kidney distal tubule epithelial MDCK cells, which were purchased from the American Type Culture Collection (ATCC; Manassas, VA, USA) were cultured in DMEM/F-12 medium supplemented with 10% fetal bovine serum (FBS) and antibiotics at 37°C in a humidified atmosphere of 95% air and 5% CO_2_. For two-dimensional culture and treatment, cells were seeded into 6-cm cell culture dishes until reaching 90% confluence, and then UCB combined with/without Z-VAD or Ferrostatin-1 were given in 1% or 10% FBS environment. For MDCK cyst culture, 10^6^ cells were resuspended with 400 μL DMEM/F-12 medium with 10% FBS, then 40 μL cell-suspension was added into 600 μL matrigel matrix. Subsequently, 50 µl matrigel matrix was dropped into the pre-cooled 6-well plate using a pre-cooled pipette tip, followed by gelling at 37°C for 1 hour, to form matrigel dome. Next, 4 mL DMEM/F-12 medium supplemented with 10% FBS was added into the well to maintain the growth and differentiation of cells. Integral cell cysts were formed after 4 ~ 5 days, which were further treated using UCB in 1% FBS environment.

### Crystal-cell adhesion assay

The COM crystals used in crystal-cell adhesion assays were prepared basing on the published protocols.^18^ Briefly, 10 mM CaCl_2_ was mixed with 1 mM NaOx in a buffer containing 90 mM Tris-HCl and 10 mM NaCl (pH 7.4). The solutions were incubated overnight at 25°C, followed by centrifugation (2000 g, 5 min). After discarding the supernatant, COM crystals were resuspended using methanol and centrifugated (2000 g, 5 min). The methanol was discarded and the COM crystals were air dried at room temperature overnight. After undergoing the indicated administration, MDCK cells were treated with 20 μg/cm^2^ COM crystals and maintained statically for 3 min. The cells were then washed three times using PBS to remove the nonadherent crystals. Subsequently, COM crystal adhesions were observed using phase‐contrast microscopy and counted.

### Cell death assay

Cell death analysis was performed using an Annexin V-FITC/PI staining detection kit. For in-situ fluorescence staining, MDCK cells were exposed to UCB in 1% FBS for 10 hours, then the medium was discarded, and MDCK cells were washed with cold PBS once, followed by incubating with Annexin V-FITC/PI mixture in the dark for 10 minutes. Next, the fluorescence was observed using the fluorescence microscope. For flow cytometric detection, MDCK cells were administered using UCB combined with/without Z-VAD or Ferrostatin-1 in 1% FBS environment for 24 hours, then cells were collected and washed three times with cold PBS, followed by incubating with Annexin V-FITC/PI mixture in the dark for 10 minutes. Next, the stained cells were determined with flow cytometry.

### Western blot analysis

MDCK cells were exposed to the indicated treatments. The whole cell protein extracts were prepared using cell lysis buffer containing protease inhibitors and phosphatase inhibitors, while the nuclear- and cytoplasmic–protein extracts were prepared using the nuclear-cytoplasm protein separation kit. Following the quantification of protein concentrations using the BCA protein assay kit, the protein samples were prepared with the addition of sample loading buffer, then subjected to SDS-PAGE separation and protein transfer to NC membranes, which were blocked using 5% nonfat milk powder in 1× PBS for 1 hour. Subsequently, the membranes were incubated overnight at 4°C with primary antibodies, followed by washing with 1× PBST three times. The membranes were then incubated with fluorescently labeled secondary antibodies (1:10000) for more than 2 hours, and the two-color infrared fluorescence protein analysis system was employed to detect the protein bands. The protein expression levels were quantified using the Image J software. The specific primary antibodies are listed as follows:

Anti-Caspase 3 (1:1000, Proteintech 66,470–2-Ig), Anti-Cleaved-caspase 3 (1:1000, CST, #9661), Anti-PARP 1 (1:5000, Proteintech 66,520–1-Ig), Anti-ZO-1 (1:1000, Proteintech 66,452–1-Ig), Anti-Occludin (1:10000, Proteintech 66,378–1-Ig), Anti-E-Cadherin (1:1000, CST, #3195), Anti-β-catenin (1:1000, CST, #8480), Anti-β-tubulin (1:1000, Proteintech 10,068–1-AP), Anti-Slc26a6 (1:50, Santa Cruz Biotechnology, sc -515,230), Anti-PKC δ (1:10000, Abcam, ab108972), Anti-Histone-H3 (1:5000, Proteintech 68,345–1-Ig), Anti-GAPDH (1:50000, Proteintech 60,004–1-Ig), Anti-β-actin (1:20000, Proteintech 66,009–1-Ig).

### Immunofluorescence assays

For the 2D-cultured cells, after the indicated treatment, MDCK cells were fixed for a period of 5 minutes using 4% formaldehyde solution. Following being washed three times using PBS, cells were permeabilized using 0.5% Triton X-100 solution for 15 minutes. For the 3D-cultured cells, after the indicated treatment, the Matrigel, which containing MDCK cysts was collected, sealed using agarose, and the cysts containing agarose cubes were fixed using 4% formaldehyde solution overnight at 4°C. After dehydrated and paraffin embedding, 4 um serial sections were produced from each paraffin block, and were hydrated to further immunofluorescent staining. For immunofluorescent staining, 4% BSA-PBS solution was employed to block nonspecific proteins for 1 hour, followed by overnight incubation with primary antibodies at 4°C. Subsequently, cells or cysts were incubated with the fluorescent secondary antibodies at room temperature for 1 hour. Next, the fluorescent mounting medium with DAPI was used to seal cells or cysts. Finally, the images of 2D-cultured cells were captured using a fluorescence inverted microscope and an imaging system (Olympus, Japan). The images of cross sections of 3D-cultured MDCK cysts were captured using a confocal fluorescent microscope, and the fluorescence intensities were quantified using the Leica Application Suite X software. The specific primary antibodies are listed as follows: Anti-ZO-1 (1:50, Proteintech 66,452–1-Ig), Anti-Occludin (1:800, Proteintech 66,378–1-Ig), Anti-E-Cadherin (1:200, Proteintech 60,335–1-Ig), Anti-β-catenin (1:200, CST, #8480), Anti-Slc26a6 (1:50, Santa Cruz Biotechnology, sc -515,230).

### Immunohistochemistry analysis (IHC)

To enhance antigen exposure, the hydrated 4-μm sections from representative animals were treated with 10 mM sodium citrate at 98°C for 20 minutes for antigen retrieval. Then, sections were incubated with an endogenous peroxidase blocking solution, followed by incubation with specific primary antibodies overnight at 4°C. Subsequently, sections were incubated with biotin-conjugated secondary antibody, followed by washing with PBS and incubating with enzyme conjugate horseradish peroxidase (HRP)-streptavidin. After HRP detection using DAB solution, the cell nucleuses was counterstained using hematoxylin. Finally, sections were mounted with aqueous mounting media. The protein expression levels were quantified using ImageJ software.

### Detection of the UCB content of kidney stones

Patient-derived stones obtained through stone extraction surgery were protected from light and grind. A small stone power of each stone sample was weighted and immersed with 200 µL DMSO, followed by sufficient ultrasonication. Then, TBIL and DBIL of the supernatant were then determined with the diazo reagent method, followed by a calculation to obtain the content of UCB.

### CaOx crystal crystallization and aggregation assay

One microliter of high concentration UCB-DMSO storage solution (1000 times) was added into 500 µl of 1 mM NaOx solution and mixed sufficiently, followed by mixing with 500 µl of 10 mM CaCl_2_·2 H_2_O solution. The mixture of CaCl_2_·2 H_2_O and NaOx with DMSO served as the control. For the nucleation assay, the alteration of turbidity with time in 20 min was measured using Cary 500 UV–Vis spectrophotometers (Varian Company, USA) below the wavelength of 620 nm. The maximum increase in optical density (OD) over time reflects the maximum rate of formation of new particles, which represents crystal nucleation. After a period of 1 hour, zeta potential ζ of the solution was detected using Malvern Zetasozer instrument. For the crystal formation assay, the mixture was incubated to avoid vibration at room temperature for 1 hour, and the amounts of formed crystals was observed using the bright field of the phase contrast microscope. For the crystal aggregation assay, the mixture was rotated up and down at room temperature for 2 hour, and the aggregation of crystals was observed using bright field of phase contrast microscope (cluster of three or more crystals was defined as aggregation).

### Metagenomic sequencing analysis of gut microbiota

#### Genomic DNA extraction

Feces were collected in sterile tubes and freeze in liquid nitrogen rapidly, then stored at −80°C. Genomic DNA of fecal samples were extracted using HiPure Bacterial DNA Kits (Magen, Guangzhou, China) according to the manufacturer’s instructions. The DNA quality was determined using Qubit (Thermo Fisher Scientific, Waltham, MA) and NanoDrop (Thermo Fisher Scientific, Waltham, MA) accordingly.

#### Illumina sequencing

Qualified genomic DNA was first fragmented by sonication to the size of 350 bp, followed by end-repairing, A-tailed and adapter-ligating using the NEBNext® Ultra™ DNA Library Prep Kit for Illumina (NEG, USA) according to the preparation protocol. Then PCR was used to enrich for 300–400 bp DNA fragments. The products of PCR were purified using the AMPure XP system (Beckman Coulter, Brea, CA, USA) and the metagenomic sequencing libraries were analyzed for appropriate size distribution using the 2100 Bioanalyzer (Agilent, Santa Clara, CA, USA) and quantified by real-time PCR. Metagenomic sequencing was performed on the Illumina NovaSeq X Plus sequencer using the pair-end technology (PE 150).

#### Quality control

Raw data from the Illumina platform were filtered using FASTP (version 0.18.0)^19^ by the following standards: 1) removing reads with ≥10% unidentified nucleotides (N); 2) removing reads with ≥50% bases having Phred quality scores ≤20; 3) removing reads aligned to the barcode adapter. After filtering, the resulting clean reads were used for genome assembly. The metagenomics profiling analysis of the 18 stool samples produced a total number of 1,306,614,094 reads. Quality and chimaera filtering resulted in 1,304,069,002 reads, with an average of 72,448,278 per sample. After filtering the host reads, a total of 1,259,255,253 clean reads have resulted for assembly.

#### Assembly, gene prediction, and gene catalogue

Clean reads of each sample were assembled individually using MEGAHIT (version 1.1.2)^20^ stepping over a k-mer range of 21 to 141 to generate sample-derived assembly. A total number of 6,254,646 contigs was assembled, and genes were predicted based on the final assembly contigs (> 500bp) using MetaGeneMark (version 3.38).^21^ The predicted genes ≥300 bp in length from all samples were pooled and combined based on ≥95% identity and 90% read coverage using CD-HIT (version 4.6)^22^ in order to reduce the number of redundant genes for the downstream assembly step. The reads were re-aligned to predict genes using Bowtie (version 2.2.5)^23^ to count read numbers. Final gene catalog was obtained from non-redundant genes with gene reads count >2.

There are three steps for gene abundance quantification: alignment, reassignment, and calculation. First, reads were aligned to unigene using Bowtie (version 2.2.5)^23^; then reads reassignment was performed using PathoScope (version 2.0.7)^24^ which provided the most sensitive profiles under common metagenomic sequencing scenarios; the relative abundance of each gene were calculated using the formula^25^:

$$G_{i}=\frac{R_{i}}{L_{i}*\sum_{j=1}^{n}\frac{R_{j}}{L_{j}}}$$

G_i_: the relative abundance of gene_i_ in sample; R: the number of mapped reads in the sample; L: the length of gene

#### Function annotation

The unigenes have been annotated with DIAMOND (version 0.9.24)^26^ by aligning with the deposited ones in diverse protein databases, including NCBI non-redundant protein (NR) database, Evolutionary Genealogy of Genes: Nonsupervised Orthologous Groups (eggNOG) database and Carbohydrate-Active Enzymes (CAZy) database. The identification of BilR relative unigenes was performed as previous description.^27^ We briefly conducted multiple sequence alignment on the identified BilR sequence of species in the GTDB,^27^ and extracted the alignment results for the first 373 amino acids. These results were then utilized for HMM training using HMMbuild to generate HMM profile. Subsequently, we performed searches using hmmsearch and only considered results with an e-value below 1e-100. The models of PF00724 and PF07992 obtained from the PFAM database were predicted based on the default threshold of HMMsearch. The predicted outcomes were intersected, and subsequently, blast was employed to align the HMM domains of the unigenes to identify potential BilR-related unigenes.

#### Taxonomic annotation

Clean reads were used to generate taxonomic profile using Kaiju (version 1.6.3).^28^ Study design of retrospective analysis. The percentage of each species contributing to the total community was shown to be in relative abundance.

#### Alpha diversity and beta diversity analysis

The Shannon index and Simpson index were calculated using Python scikit-biopackage^29^ (version 0.5.6) and used to assess alpha diversity. Alpha index comparisons among groups were computed using the Kruskal–Wallis H test in the R project Vegan package.^29^ Bray-Curtis distances based on gene and function abundances were utilized to determine beta diversity. PCoA (Principal Coordinates Analysis) of Bray-Curtis distances was calculated using R vegan package^29^ and plotted using R ggplot2 package.^30^

#### Difference analysis

The comparison of the unigenes/species relative abundances between the two groups was calculated using the Wilcoxon rank test, and a comparison among the three groups was computed by ANOVA (analysis of variance) in R project Vegan package.^29^ Multiple testing corrections were performed to calculate the q-value. Through the LEfSe software (version 1.0),^31^ linear discriminant analysis effect size (LEfSe) was used to visualize the differentially abundant species that exhibited significant differences in abundance among samples from different groups, with linear discriminant analysis (LDA) scores exceeding 2.5 within each group. Enrichment Ternary Plot was used to present significantly different species. The R package ggtern was used to draw species enrichment ternary plots.

### Fecal sample DNA extraction and quantitative PCR for microbiota analysis

Feces were collected in sterile tubes and freeze in liquid nitrogen rapidly, then stored at −80°C. DNA was extracted from 250 mg fecal sample using TIANamp Stool DNA Kit (TIANGEN, Beijing, China), following the manufacturer’s instructions. The concentrations of gDNA were then quantified to 1ng/μL. The abundance of the indicated unigenes/species was quantified using a Bio-Rad CFX96 system with SYBR Green (TaKaRa, Tokyo, Japan). Total 16S rDNA was served as an internal control. The specific primer sequences are listed in Supplementary materials (Table S4. table of primers sequences).

### β-GD activity assay

Fecal samples were homogenized, and an appropriate volume of saline was added to achieve a concentration of 0.05 g/mL. Following vortexing and shaking, the samples were subjected to centrifugation (500 rpm, 5 min). Thereafter, samples were split by ultrasonic (placed on ice, 200 W, work time 3 s, interval 10 s, total time 3 min) and were performed, followed by centrifugation (10,000 rpm, 20 min, 4 ℃). The supernatant was collected and placed on the ice for testing. The β-GD activity of the samples was detected and calculated using β-Glucuronidase (β-GD) Activity Assay Kit (SUNLON, Hangzhou, China), according to the manufacturer’s instructions.

### RNA extraction and RT-qPCR analysis

For the measurement of mRNA expression of cultured cells, total RNAs were isolated from cells using Trizol reagent according to the manufacturer’s instructions. Subsequently, 1 µg of total RNA was subjected to reverse transcription using PrimeScript™ RT reagent Kit (TaKaRa, Tokyo, Japan). Quantitative PCRs were conducted using a Bio-Rad CFX96 system with SYBR Green (TaKaRa, Tokyo, Japan) to determine the mRNA expression of *Slc26a6*. The expression levels were normalized to the expression of *β-tubulin* or *GAPDH*.

For the measurement of mRNA expression of Malpighian tubules, Malpighian tubules were collected and washed, then total RNAs were isolated and mRNA expression of *Pristin* mRNA was detected using the same protocol as for the cultured cells. The expression levels were normalized to the expression of *RpL32* mRNA.

For the measurement of mRNA expression of gut microbiota, total RNA was extracted from 300 mg fecal samples using fecal RNA isolation kit (Bioteke, Beijing, China) following the manufacturer’s instructions. Total RNA was subjected to an rRNA removal procedure using the Ribo-Zero Plus Microbiome rRNA Depletion Kit according to the manufacturer’s instructions (Illumina, Shanghai, China). Then RNA was transcribed into DNA using PrimeScript™ RT reagent Kit (TaKaRa, Tokyo, Japan) for qPCR. Representative *BilR* unigenes sequences were used to deduce primer pairs. The specific primer sequences are listed in Supplementary materials (Table S4. table of primers sequences).

### Study design of retrospective analysis

A retrospective data collection was conducted from the Second Affiliated Hospital of Guangzhou Medical University from March 2016 to July 2020. Patients with tumor, renal function impairment, glomerulonephritis, gout, hepatocirrhosis, fever, hypertension and diabetes, and with uncertainty diagnosis of urinary stones due to the lack of urinary imaging were excluded. Then, we further excluded patients with hyper-total bilirubin ( > 21 umol/L) and urinary stone which mainly composed of non-CaOx. Finally, we collected data from 2156 eligible individuals. Individuals without any stones as identified by urinary ultrasonography were designated as the Control group, and based on the composition analysis of stones, patients with stone which mainly composed of CaOx were designated as CaOx-stone group. The laboratory information was obtained from the hospital information system, including serum creatinine (Scr, µmol/L), alanine aminotransferase (ALT, U/L), serum total protein (TP, g/L), total bile acid (TBA, umol/L), γ-glutamyl transpeptidase (GGT, U/L), total cholesterol (TC, mmol/L), triglyceride (TG, mmol/L), high-density lipoprotein cholesterol (HDL-C, mmol/L), low-density lipoprotein cholesterol (LDL-C, mmol/L), uric acid (UA, umol/L), total bilirubin (umol/L), direct bilirubin (DBIL, umol/L), indirect bilirubin (UCB, umol/L), albumin (Alb, g/L), white blood cell (WBC, 10^9/L), neutrophil count (NEUT#, 10^9/L), lymphocyte count (LYMPH#, 10^9/L), monocyte count (MONO#, 10^9/L), red blood cell (RBC, 10^12/L), hemoglobin (Hb, g/L), platelet (PLT, 10^9/L), urine pH, urine ketone bodies (KET), urine-specific gravity (SG), urine protein (PRO), urobilinogen (URO), urine nitrite (NIT). Our study design was reviewed and approved by the ethics committee of the Second Affiliated Hospital of Guangzhou Medical University (NO). 2024-hg-ks-43). The requirement for informed consent was waived, as all the information was retrospectively extracted from medical records.

### Statistical analysis

Statistical analyses were conducted using RStudio (Version 4.4.0) and GraphPad Prism (Version 9.1.1) software. When the data were normally distributed, differences between two groups were assessed using t-tests, among more than two groups were assessed using one-way ANOVA tests with Bonferroni’s multiple comparisons; Correlations were analyzed by computing Pearson correlation coefficients. When the data were non-normally distributed, differences between two groups were assessed using nonparametric tests, among more than two groups were assessed using Kruskal–Wallis tests with Dunn’s multiple comparisons; Correlations were analyzed by computing nonparametric Spearman correlation. For the statistical analysis of clinical retrospective data, the continuous variables exhibiting a normal distribution were described as mean ± standard deviation and compared using t-tests; the continuous variables exhibiting a non-normal distribution were described as median (1st Quartile, third Quartile) and compared using Mann–Whitney tests. Categorical variables were described as frequencies (percentages) and compared using chi-square tests. The odds ratios (OR) and the 95% confidence intervals (CI) for the association between variables and CaOx stone were calculated using Binary Logistic Regression Analysis. Statistical tests used for group and cluster comparisons in metagenomics and metabolomics were specified in the respective sections in methods. A *p* value <0.05 was considered statistical significant for all data sets. *P* values were represented on figures as follows: ns, not significant, *p < 0.05, **p < 0.01, ***p < 0.001, ****p < 0.0001.

## Results

### Intestinal UCB is associated with healthy-FMT regulated renal CaOx crystal deposition in rats

**Figure 1.**
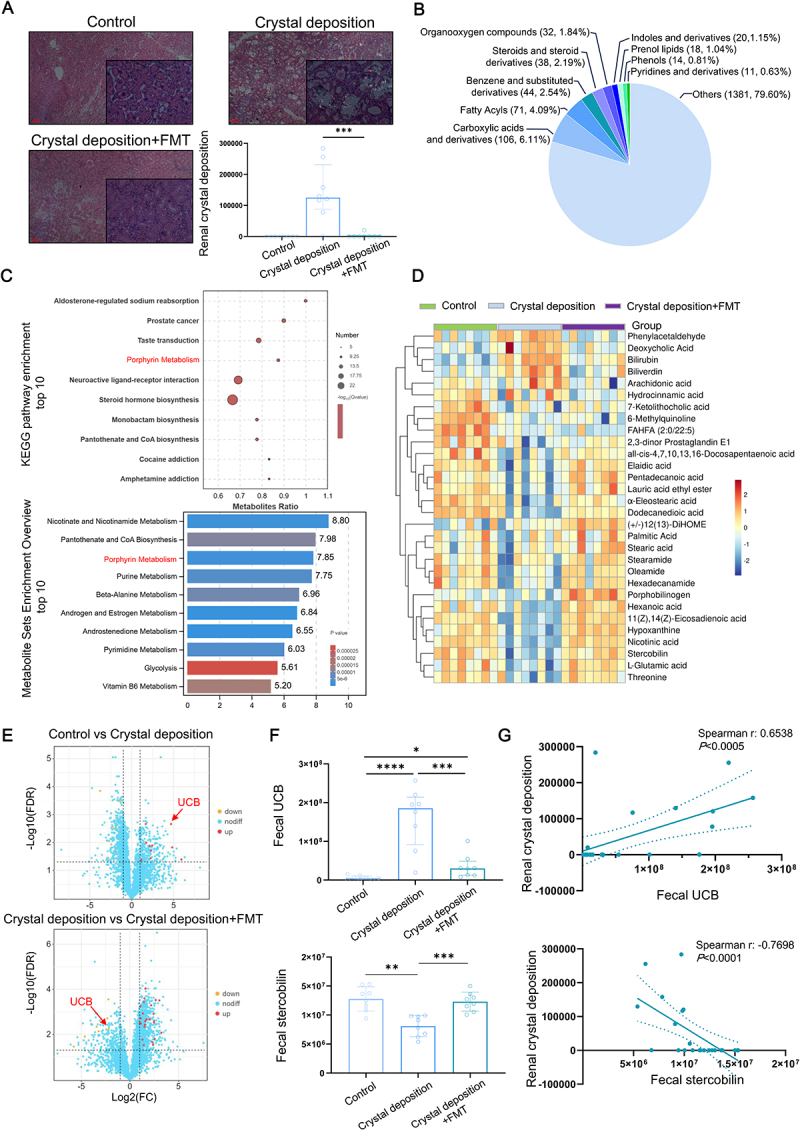
UCB is an intestinal metabolite associated with healthy-FMT-regulated renal CaOx crystal deposition in rats. (A) the CaOx crystal deposition in rat kidneys was observed using polarizing microscope, and the amount of crystal-containing tubules was used to evaluating the level of renal crystal deposition. (bar = 100 μm; quantitative data shown are median with interquartile range, *n* = 8 of each group). (B-E) the fecal metabolomics profiling was conducted using UHPLC-MS/MS analyses. Combined with statistical measures like *p* < 0.05, the category statistics pie chart showing the composition ratio and the number in each class of identified differential metabolites between Control, crystal deposition and crystal deposition+FMT group (B); the bubble chart showing top 10 (FDR) enriched pathway based on KEGG analysis and the bar chart showing the top 10 (FDR) enriched pathway based on metabolite Set enrichment analysis (MSEA) (C); the heat map illustrating differential profile of fecal metabolites (D); combined with statistical measures like VIP ≥ 2, FDR < 0.05, Fold change ≥ 2 to select key differential metabolites, the volcano plot showing differential fecal metabolites between indicated groups (E); (F) the content of fecal UCB (quantitative data shown are median with interquartile range) and stercobilin (quantitative data shown are mean with SD) between indicated groups (*n* = 8 of each group). (G) the correlation analysis between the fecal UCB and renal crystal deposition, and the correlation analysis between the fecal stercobilin and renal crystal deposition (*n* = 16). * *p* < 0.05, **p < 0.01, ***p < 0.001, ****p < 0.0001.

Metagenomic sequencing was used to analyze the alternation of gut microbial composition and functional genes, in order to assess the ability of healthy-FMT to reconstitute gut microbiota first. Alpha diversity measures, including Shannon and Simpson index at species level, were assessed to evaluate the richness and evenness of microbial communities. The alpha diversity of the gut microbiota in the Crystal deposition group was observed to be lower compared to the Control group, which was significantly restored by healthy-FMT (Figure S1A). Beta diversity was investigated using Bray-Curtis and principal coordinate analysis (PCoA), and the results demonstrated distinct clustering of gut microbial composition at the species level among the three groups (Figure S1B). Furthermore, as shown in Figure S1C, the beta diversity analysis revealed significant differences in gene function composition of gut microbiota among the three groups. We then assessed the potential impact of gut microbial reconstitution using healthy-FMT in reducing HYP-induced experimental renal crystal deposition in rats. As shown in Figure 1A, healthy-FMT effectively mitigates HYP-induced renal CaOx crystal deposition. These results reveal a significant disruption in the gut microbiota of crystal deposition rats, and the powerful role of healthy FMT in reconstructing gut microbiota and inhibiting renal crystal deposition.

Intestinal metabolites can be absorbed through the intestinal wall and influence distant organs via circulatory systems, facilitating communication between altered gut microbiota and target organs. To identify key metabolites that transmit signals from the gut to the kidneys, potentially mediating CaOx crystal formation, we analyzed fecal metabolomics profiling using UHPLC-MS/MS.

Combined with statistical measures like *p* < 0.05, we identified 1735 differential metabolites between Control, Crystal Deposition and Crystal deposition + FMT group, encompassing Carboxylic acids and derivatives, fatty acids, Benzene, and substituted derivatives, Steroids, and steroid derivatives, Organooxygen compounds, Indoles, and derivatives, Prenol lipids, Phenols, Pyridines and derivatives, and other compounds (Figure 1B). The results of pathway enrichment analysis revealed that the Porphyrin Metabolism pathways were significantly influenced (Figure 1C). The heatmap shows alterations in the content of differential metabolites, including metabolites such as UCB in the porphyrin metabolic pathway (Figure 1D). Combined with statistical measures like VIP ≥ 2, FDR < 0.05, fold change ≥2 to select key differential metabolites, volcano plot analysis revealed that fecal UCB was significantly increased in the Crystal deposition group when compared to the Control group; however, UCB was significantly diminished in the Crystal deposition-FMT group in comparison to the Crystal deposition group (Figure 1E). Fecal Stercobilin is one of the final metabolites of fecal UCB, as a downstream product of UCB reduction. Results of multiple comparisons not only verified changes in fecal UCB levels but also revealed that fecal stercobilin was reduced in the Crystal deposition group and restored following health-FMT (Figure 1F). Notably, we observed a positive correlation between fecal UCB levels and renal crystal deposition, and a negative correlation between fecal stercobilin levels and renal crystal formation (Figure 1G). These findings suggest that UCB accumulation and impaired conversion to stercobilin are linked to the gut microbiota dysbiosis observed in renal crystal deposition rats, and that reconstituting healthy gut microbiota by healthy-FMT can reverse this metabolic disruption.

### Circulating UCB is associated with renal CaOx crystal deposition in rats

**Figure 2.**
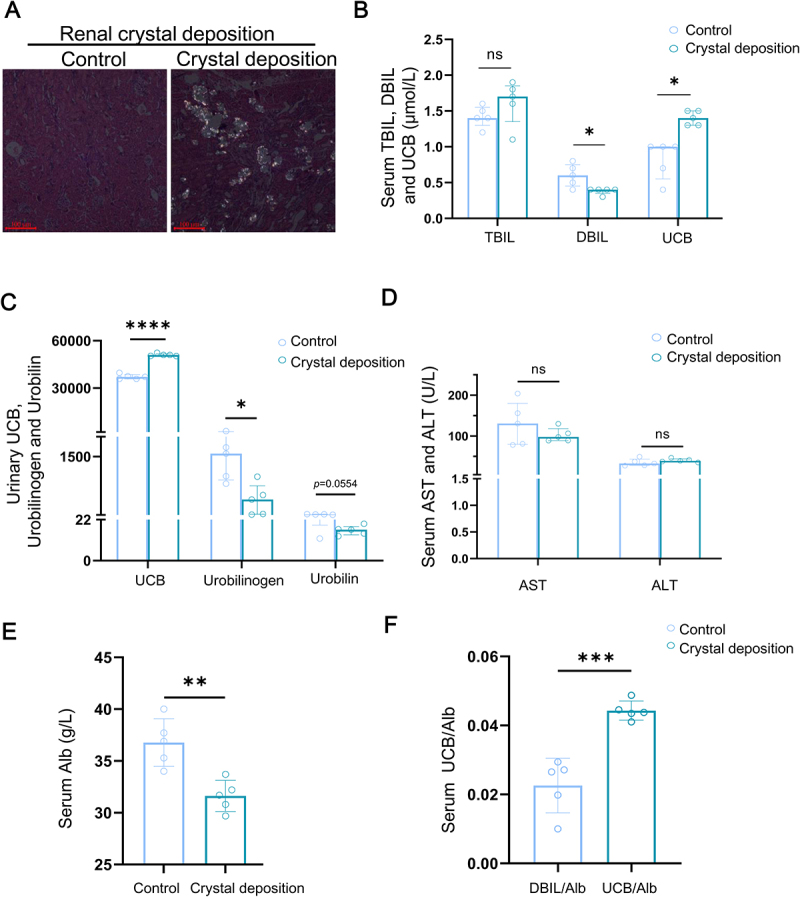
Circulatory bilirubin activity of renal CaOx crystal deposition rat is stronger than the Control group. (A) the renal CaOx crystal deposition was observed using polarizing microscope (bar = 100 μm). (B) the levels of circulatory bilirubin activity-relative indexes, including total bilirubin, DBIL and UCB, were detected using standard chemical methods (quantitative data shown are median with interquartile range, *n* = 5 of each group). (C) the levels of serum indicators of liver function, including AST and ALT, were detected using standard chemical methods (quantitative data shown are median with interquartile range, *n* = 5 of each group). (D) the levels of urinary UCB, urobilinogen and urobilin were evaluated using LC-MS analysis (quantitative data shown are median with interquartile range, *n* = 5 of each group). (E) the levels of serum Alb was detected using standard chemical methods. (quantitative data shown are mean with SD, *n* = 5 of each group). (F) the levels of UCB/Alb were calculated (quantitative data shown are median with interquartile range, *n* = 5 of each group). ns. no significant, * *p* < 0.05, **p < 0.01, ***p < 0.001, ****p < 0.0001.

To further investigate alterations in circulating UCB, we examined serum and urinary biochemical indices related to UCB in another renal CaOx crystal deposition rat model. As shown in Figure 2A, EG administration induced obvious renal CaOx crystal deposition. Both serum and urinary UCB levels were elevated in the Crystal deposition group (Figure 2B,C). In contrast, there were no significant differences in liver function, indicated by levels of AST and ALT (Figure 2D). We also analyzed urinary metabolites associated with UCB. Urinary UCB level was significantly higher in the Crystal deposition group, whereas urinary levels of urobilinogen and urobilin, which are metabolized from intestinal UCB and urinary excreted after intestinal absorption, were significantly lower than in the Control group (Figure 2C).

UCB, being lipid-soluble can readily cross cell membranes and exert intracellular effects. However, its lipid solubility decreases significantly when bound to albumin (Alb) or when converted into CB. Therefore, the UCB/Alb ratio is considered a more reliable indicator of circulating bilirubin activity in target organs than total bilirubin, UCB, or DBIL levels alone. Our biochemical analysis showed that both serum DBIL and Alb were reduced in the Crystal deposition group, while the UCB/Alb ratio was higher compared to controls (Figure 2B–F). These findings suggest elevated systemic UCB activity in rats with renal CaOx crystal deposition.

### UCB is linked to clinical CaOx stone formation in humans

Renal crystal deposition is one of the most important factors that initiate the clinical development of CaOx stones. To elucidate the changes in serum UCB activities in humans, we further conducted a retrospective analysis. Before matching of gender and age, results shown that serum levels of total bilirubin and UCB were higher in the CaOx-stone group compared to controls. Conversely, serum levels of DBIL and Alb were lower in the CaOx-stone group (Figure S2A–D). The UCB/Alb ratio was elevated in the CaOx-stone group compared to controls (Figure S2E), while the DBIL/Alb ratio was lower in the CaOx-stone group (Figure S2F). Table S1 provides a summary of the gender, age, and other clinical indexes of the study population based on CaOx stone status. A further logistic regression analysis demonstrated that each unit increase in the UCB/Alb ratio (adjusted by a factor of 100) was associated with a 7% higher risk of CaOx stones (Table S2).

**Figure 3.**
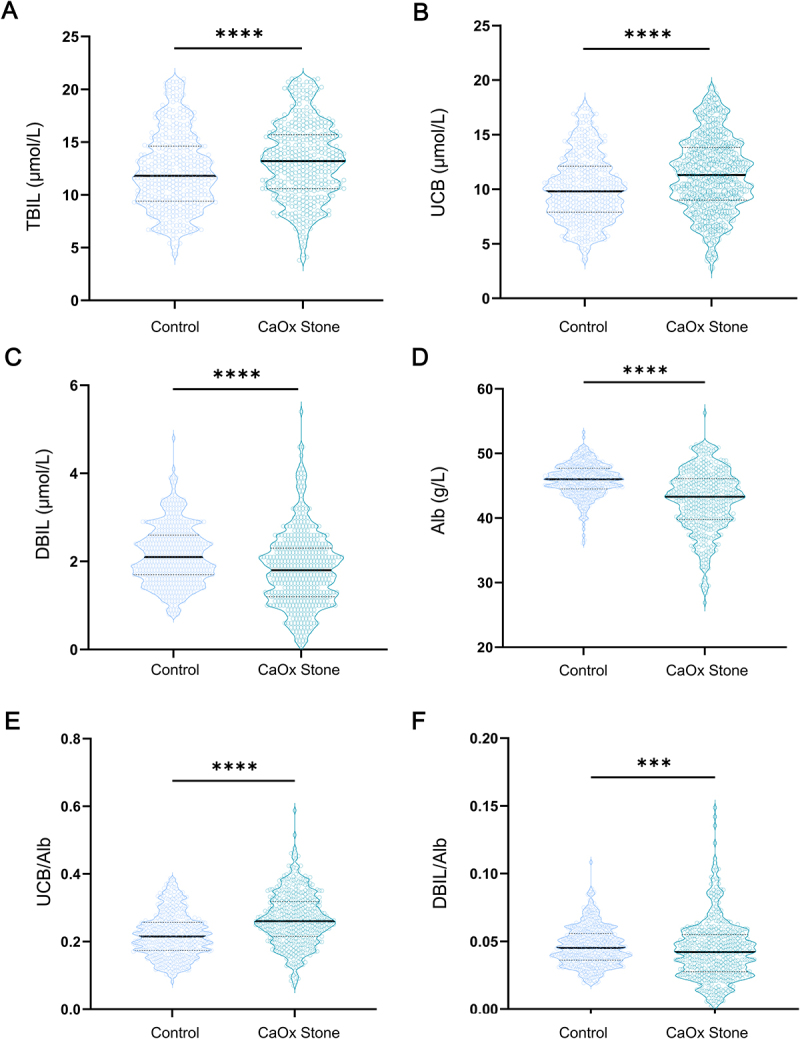
The comparison of circulatory bilirubin activity-relative laboratory indexes between the Control group and the CaOx-stone patient group after matching of gender and age. (A-D) the levels of serum total bilirubin (A), UCB (B), DBIL (C) and Alb (D) were evaluated using standard chemical methods (quantitative data shown are median with interquartile range). (E-F) the levels of UCB/Alb ratio (E); DBIL/Alb ratio (F) were calculated (quantitative data shown are median with interquartile range). *n* = 427 of each group, ***p < 0.001, **p < .0001.

To minimize potential confounding by gender and age, a matched cohort of 427 CaOx-stone patients and 427 controls was analyzed. The trends in serum total bilirubin, UCB, DBIL, Alb and DBIL/Alb ratio were consistent with the results before matching (Figure 3A–D,F). Notably, the UCB/Alb ratio remained significantly elevated in the CaOx-stone group (Figure 3E). Additional clinical indexes summarized according to stone status are detailed in Table S3. Univariate and multivariate logistic regressions analyses, presented in Table 1, identified the UCB/Alb ratio (adjusted by a factor of 100) as a significant risk factor for CaOx stones. In univariate analysis, the odds ratio (OR) was 1.10 (95% CI: 1.08–1.12, *p* < 0.001), and in multivariate analysis, the OR was 1.21 (95% CI: 1.15–1.29, *p* < 0.001).

**Table 1.** Univariate and multivariate analysis for analyzing the risk factors of CaOx stones after matching of gender and age.

Variables	Univariate Unadjusted						Multivariate Adjusted				
	β	S.E	Z	*P*	OR (95%CI)		β	S.E	Z	*P*	OR (95%CI)
KET											
Negative					1.00 (Reference)						
Trace	1.83	1.05	1.74	0.081	6.22 (0.80 ~ 48.44)						
Positive	0.82	1.12	0.73	0.467	2.26 (0.25 ~ 20.36)						
SG											
≤1.005					1.00 (Reference)						
1.005–1.030	−0.15	0.21	−0.72	0.474	0.86 (0.58 ~ 1.29)						
≥1.030	0.52	0.47	1.11	0.269	1.68 (0.67 ~ 4.21)						
PRO											
Negative					1.00 (Reference)						1.00 (Reference)
Positive	1.57	0.23	6.86	** <.001**	4.78 (3.06 ~ 7.48)		0.95	0.44	2.15	**0.031**	2.60 (1.09 ~ 6.19)
URO											
Negative					1.00 (Reference)						
Positive	1.84	1.05	1.75	0.080	6.27 (0.80 ~ 48.81)						
NIT											
Negative					1.00 (Reference)						1.00 (Reference)
Positive	3.43	1.01	3.38	** <.001**	30.83 (4.23 ~ 224.76)		3.03	1.17	2.59	**0.009**	20.66 (2.10 ~ 203.64)
Scr (umol/L)	0.03	0.00	7.38	** <.001**	1.03 (1.02 ~ 1.04)		0.06	0.01	5.30	** <.001**	1.07 (1.04 ~ 1.09)
ALT (U/L)	−0.00	0.00	−0.45	0.651	1.00 (0.99 ~ 1.00)						
TP (g/L)	−0.13	0.02	−8.38	** <.001**	0.88 (0.85 ~ 0.90)		−0.14	0.04	−3.34	** <.001**	0.87 (0.81 ~ 0.95)
TBA (umol/L)	−0.01	0.01	−0.63	0.531	0.99 (0.97 ~ 1.01)						
GGT (U/L)	−0.00	0.00	−1.47	0.141	1.00 (0.99 ~ 1.00)						
TG (mmol/L)	0.29	0.06	4.41	** <.001**	1.33 (1.17 ~ 1.51)						
HDL-C (mmol/L)	−1.59	0.26	−5.99	** <.001**	0.20 (0.12 ~ 0.34)		−1.27	0.52	−2.43	**0.015**	0.28 (0.10 ~ 0.78)
LDL-C (mmol/L)	−0.69	0.11	−6.14	** <.001**	0.50 (0.40 ~ 0.62)		−0.73	0.23	−3.16	**0.002**	0.48 (0.31 ~ 0.76)
UA (umol/L)	0.00	0.00	1.76	0.078	1.00 (1.00 ~ 1.00)						
WBC (10^9/L)	0.36	0.05	7.80	** <.001**	1.44 (1.31 ~ 1.58)						
NEUT# (10^9/L)	0.61	0.06	9.46	** <.001**	1.84 (1.62 ~ 2.09)		0.82	0.16	5.25	** <.001**	2.28 (1.68 ~ 3.10)
LYMPH# (10^9/L)	−0.19	0.11	−1.75	0.080	0.83 (0.67 ~ 1.02)						
MONO# (10^9/L)	−2.45	0.50	−4.95	** <.001**	0.09 (0.03 ~ 0.23)		−9.47	1.70	−5.58	** <.001**	0.00 (0.00 ~ 0.00)
RBC (10^12/L)	0.58	0.12	4.89	** <.001**	1.78 (1.41 ~ 2.25)		1.09	0.32	3.40	** <.001**	2.99 (1.59 ~ 5.61)
Hb (g/L)	−0.02	0.00	−4.69	** <.001**	0.98 (0.97 ~ 0.99)		−0.03	0.01	−2.52	**0.012**	0.97 (0.95 ~ 0.99)
PLT (10^9/L)	0.01	0.00	2.37	**0.018**	1.01 (1.01 ~ 1.01)		0.01	0.00	3.27	**0.001**	1.01 (1.01 ~ 1.02)
Urine pH	0.57	0.15	3.81	** <.001**	1.77 (1.32 ~ 2.36)		0.92	0.25	3.67	** <.001**	2.50 (1.53 ~ 4.08)
DBIL/Alb (100times)	−0.08	0.04	−2.12	**0.034**	0.92 (0.86 ~ 0.99)		−0.75	0.13	−5.90	** <.001**	0.47 (0.37 ~ 0.61)
UCB/Alb (100times)	0.09	0.01	8.77	** <.001**	1.10 (1.08 ~ 1.12)		0.19	0.03	6.59	** <.001**	1.21 (1.15 ~ 1.29)
OR: Odds Ratio, CI: Confidence Interval											

Scr, serum creatinine; ALT, alanine aminotransferase; TP, serum total protein; TBA, total bile acid; GGT, γ-glutamyl transpeptidase; TG, triglyceride; HDL-C, high density lipoprotein cholesterol; LDL-C, low density lipoprotein cholesterol; UA, uric acid; WBC, white blood cell; NEUT#, neutrophil count; LYMPH#, lymphocyte count; MONO#, monocyte count; RBC, red blood cell; Hb, hemoglobin; PLT, platelet; KET, urine ketone bodies; SG, urine specific gravity; PRO, urine protein; URO, urobilinogen; NIT, urine nitrite.

Collectively, these findings demonstrate a positive association between serum UCB activity and CaOx stone formation in humans.

### UCB exacerbates renal crystal deposition in Drosophila and rats

**Figure 4.**
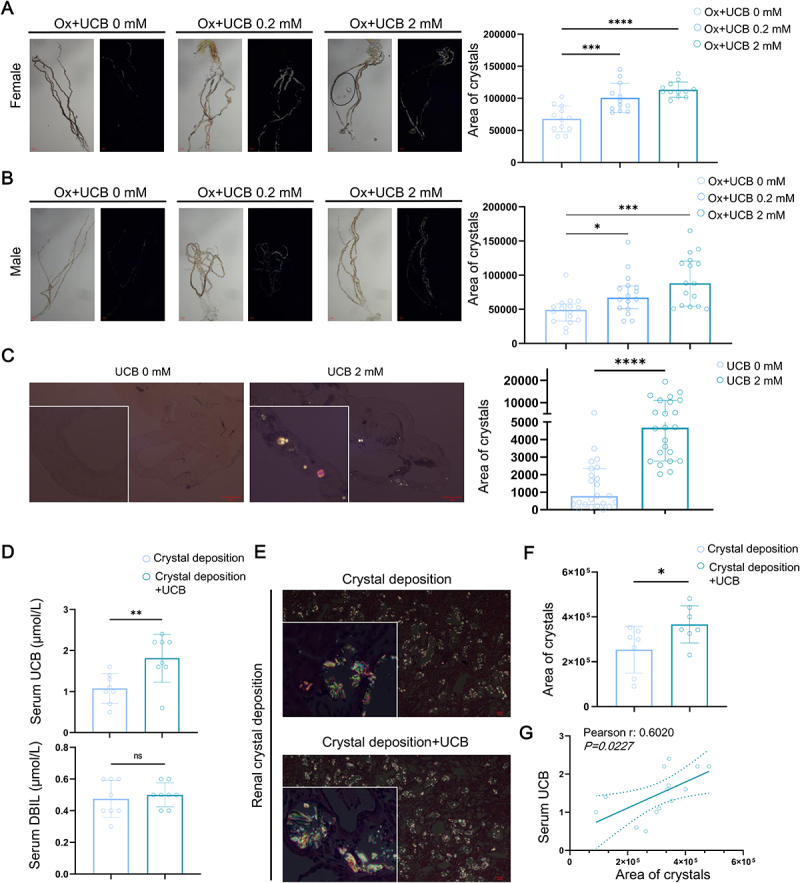
UCB exacerbates renal crystal retention *in vivo*. (A-B) The renal CaOx crystal deposition in Malpighian tubules of hyper-Ox diet fly was evaluated using polarizing microscope (bar = 100 μm; left: bright field; right: polarizing field). (A: female fly; quantitative data shown are mean with SD, *n* = 12 of each group). (B: male fly; quantitative data shown are median with interquartile range, *n* = 16 of each group). (C) The renal CaOx crystal deposition in Malpighian tubules of normal diet fly was evaluated using polarizing microscope (bar = 100 μm; quantitative data shown are median with interquartile range, *n* = 23 of each group). (D) The levels of serum UCB and DBIL of rat were evaluated using standard chemical methods (quantitative data shown are mean with SD, *n* = 8 of each group). (E-F) The renal CaOx crystal deposition of rat were evaluated using polarizing microscope (bar = 100 μm) (E); the bar chart showing the serum UCB of rat between indicated groups (quantitative data shown are mean with SD, *n* = 7 of each group (F). (G) The correlation analysis between the serum UCB and renal crystal deposition (*n* = 14). ns. no significant, * *p* < 0.05, **p < 0.01, ***p < 0.001, ****p < 0.0001.

*Drosophila* is an effective model for studying renal CaOx crystal deposition because it is biologically conserved and easy to maintain. Polarized light microscopy of the Malpighian tubules, which function analogously to mammalian kidneys, revealed that UCB intake significantly increased Oxalate-induced-CaOx crystal retention in female flies (Figure 4A). Similarly, UCB intake also elevated oxalate-induced-CaOx crystal retention in male flies (Figure 4B), further supporting a sex-independent effect of UCB on crystal accumulation. In addition, compared to the Control group, more spontaneous CaOx crystal deposition was observed in normal feeding female flies after 27 days of 2 mM UCB administration (Figure 4C).

To validate these observations in a mammalian system, we extended our investigation to a rat model of renal CaOx crystal deposition. UCB administration led to a marked elevation in serum UCB levels, yet had no significant impact on serum DBIL levels (Figure 4D); meanwhile, no significant differences of body weight, urine output, or markers of renal and liver function were detected between the Crystal deposition and the Crystal deposition + UCB group (Figure S3), suggesting that UCB organ dysfunction induced broader systemic toxicity or organ dysfunction. More importantly, UCB treatment enhanced renal CaOx crystal deposition (Figure 4E,F), paralleling the findings observed in *Drosophila*. Furthermore, a strong positive correlation between serum UCB levels and renal CaOx crystal retention (Figure 4G) underscores UCB’s potential role as a key modulator in the renal CaOx crystal deposition.

Overall, these results provide evidence that elevated UCB levels contribute to the pathogenesis of renal CaOx crystal deposition.

### UCB promotes CaOx crystal adhesion to the renal tubular epithelial cells

**Figure 5.**
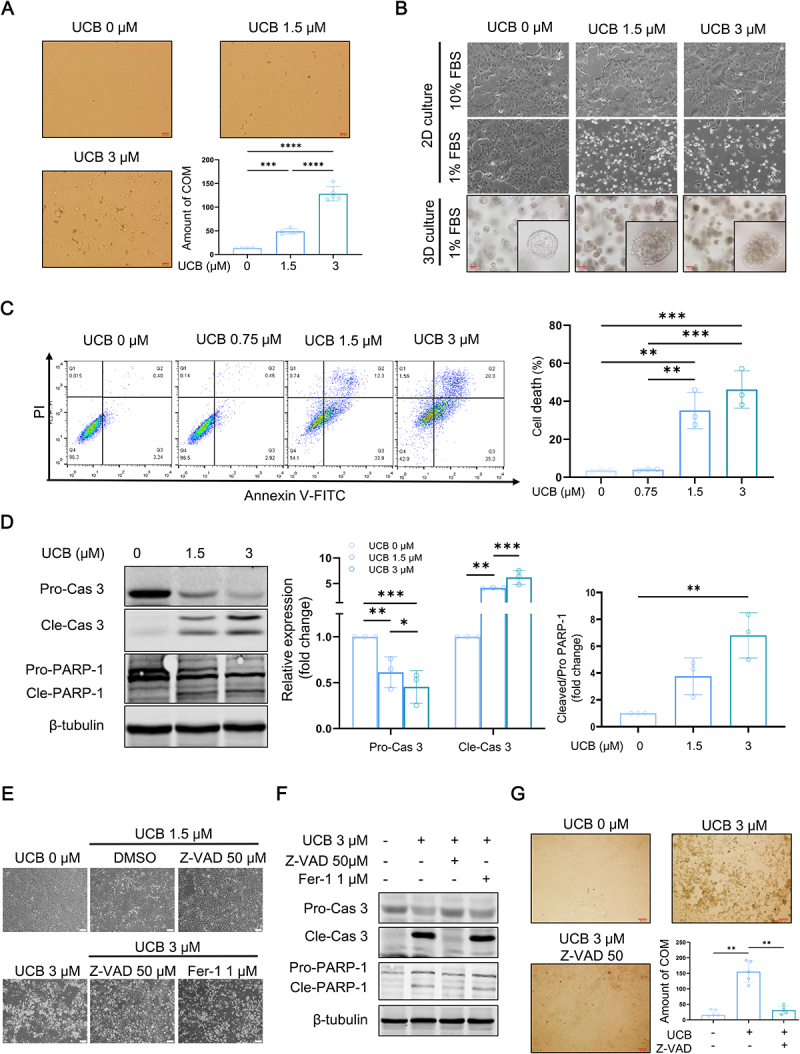
UCB promotes CaOx crystals adhesion to the renal tubular epithelial MDCK cells. (A) MDCK cells were treated with UCB in 1% FBS environment for 8 hours, followed by treatment of COM crystals. The amount of COM crystals adhered to cells was counted (bar = 20 μm; quantitative data shown are mean with SD, *n* = 5 of each group). (B) 2D cultured- and 3D cultured MDCK cells were treated with UCB and the morphology of cell was observed using phase contrast microscope (2D-Bar = 200 μm, 3D-Bar = 50 μm). (C) MDCK cells were treated with UCB in 1% FBS environment for 24 hours. Cell death was detected using Annexin V-FITC and PI staining and flow cytometric detection (quantitative data shown are mean with SD, *n* = 3 of each group). (D) MDCK cells were treated with UCB in 1% FBS environment for 24 hours. Western blotting analysis was performed to assess the expression of the indicated proteins. Pro-Cas3: pro-caspase 3; Cle-cas 3: cleaved-caspase 3; Cle-PARP-1: cleaved-PARP-1; (quantitative data shown are mean with SD, *n* = 3 of each group). (E) MDCK cells were treated with UCB in 1% FBS environment, combining with/without Z-VAD or fer-1 for 24 hours. The morphology of MDCK cells were observed using phase contrast microscope (bar = 50 μm). (F) MDCK cells were treated with UCB in 1% FBS environment, combining with/without Z-VAD or fer-1 for 24 hours. Western blotting analysis was performed to assess the expression of the indicated proteins. (G) MDCK cells were treated with UCB in 1% FBS environment combining with/without Z-VAD for 8 hours, followed by treatment of COM crystals. The amount of COM crystals adhered to cells was counted (bar = 50 μm; quantitative data shown are median with interquartile range, *n* = 5 of each group). **p < .05, ***p < 0.01, ****p < 0.001, **p < 0.0001.

The mechanism by which UCB aggravates CaOx crystal formation was explored *in vitro*. A crystal-cell adhesion assay demonstrated that UCB significantly increased the adhesion of calcium oxalate monohydrate (COM) crystals to MDCK renal tubular epithelial cells under hypo-FBS conditions (Figure 5A). Since cell death is a known trigger for crystal adhesion, we examined whether UCB induced cell death-like morphological changes. UCB causes death-like changes in MDCK cells and structural damage to three-dimensional cysts formed by MDCK cells (Figure 5B). Cytotoxicity was confirmed by Annexin V-FITC/PI staining and flow cytometry (Figure S4A and Figure 5C).

Further analysis revealed that UCB-induced cell death involved both DNA and mitochondrial damage (Figure S4B and S4C). UCB also activated apoptosis-related proteins, including caspase-3 and its substrate poly [ADP-ribose] polymerase 1 (PARP-1) (Figure 5D), and Z-VAD-FMK, a caspase inhibitor, significantly reduced UCB-induced cell death, inhibited cleavages of caspase-3 and PARP-1 (Figure 5E,F and Figure S4D). We also investigated the potential involvement of ferroptosis, a form of lipid peroxidation-mediated cell death, implicated in UCB-induced cell death. However, treatment with ferrostatin-1, a selective ferroptosis inhibitor, did not prevent UCB-induced cell death, confirming that apoptosis, rather than ferroptosis, is the predominant form of cell death induced by UCB (Figure 5E,F and Figure S4D).

Additionally, tight junction disruption, another factor contributing to COM crystal adhesion, was evaluated. UCB treatment reduced the expression of key tight junction proteins, including zonula occludens-1 (ZO-1), occludin, and the E-cadherin/β-catenin complex, which could be restored by apoptosis inhibition (Figure S5). Importantly, Z-VAD-FMK treatment significantly decreased COM crystal adhesion, further indicating that UCB-induced apoptosis mediates cell-crystal adhesion (Figure 5G).

### UCB enhances transmembrane secretion of oxalate in renal tubular epithelial cells

**Figure 6.**
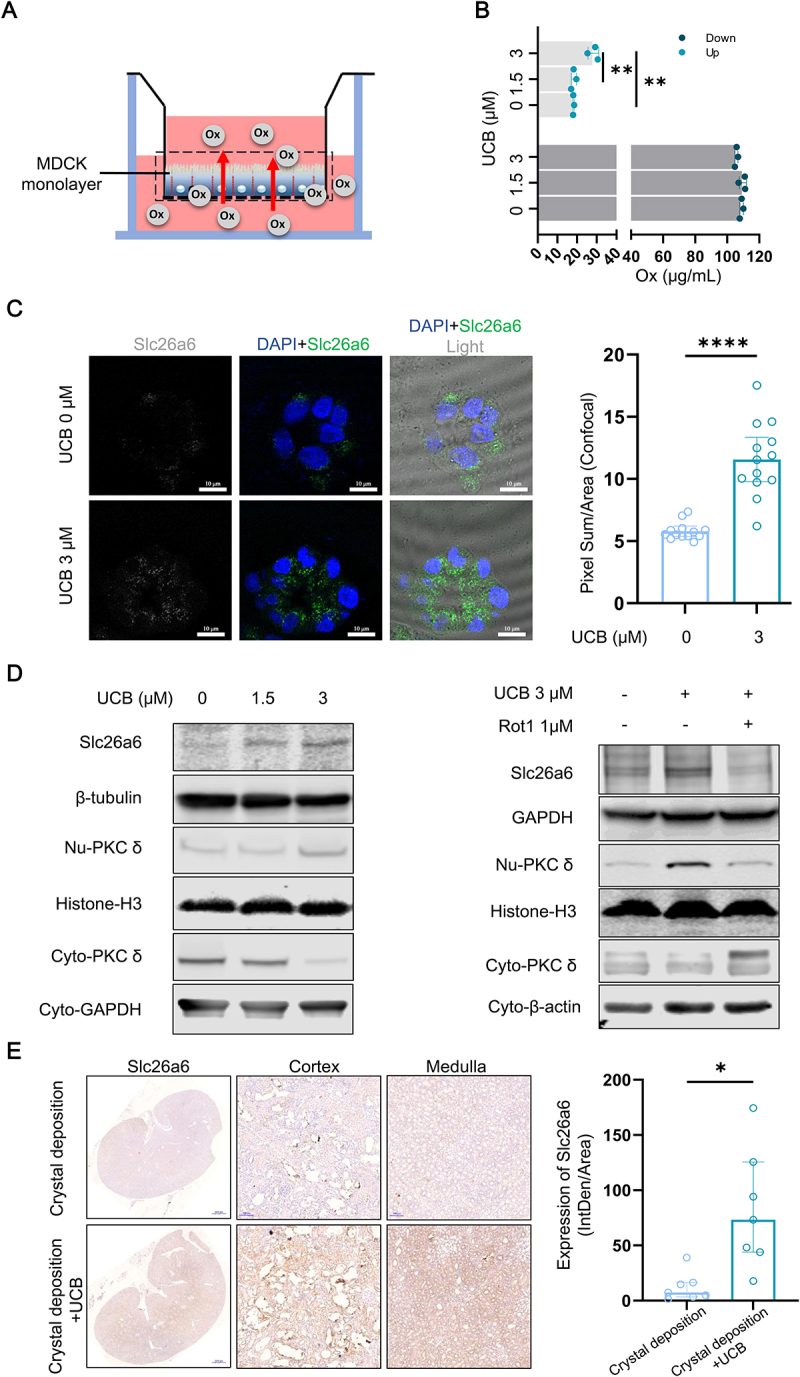
UCB increases Slc26a6 expression of renal tubular epithelial cells and promotes transmembrane oxalate excretion. (A) The diagram illustrates an in vitro cell model that simulates renal tubular epithelium and the detection of oxalate secretion. (B) MDCK cells were treated with UCB in 1% FBS environment for 10 hours, followed by oxalate treatment in basal membrane side for 2 hours. The oxalate concentrations of upper medium and lower medium were detected using ion exchange chromatography (quantitative data shown are mean with SD, *n* = 3 of each group). (C) 3D-cultured cysts of MDCK cells were treated with UCB in 1% FBS environment for 6 hours. The expression of Slc26a6 was detected using if (confocal) (bar = 10 μm; quantitative data shown are median with interquartile range, *n* = 13 of each group). (D) MDCK cells were treated with UCB in 1% FBS environment for 6 hours, combining with/without rottlerin. The expressions of indicate proteins were detected using Western blotting analysis. (E) the renal tubular Slc26a6 expression of rat was evaluated using IHC analysis (bar = 100 μm; quantitative data shown are median with interquartile range, *n* = 7 of each group). * *p* < 0.05, **p < 0.01, ***p < 0.0001.

Given that hyperoxaluria is a key risk factor for renal CaOx crystals formation, originating from glomerular filtration and tubular secretion, the effect of UCB on tubular oxalate secretion was investigated. MDCK cells were cultured in a permeable insert to simulate the renal collecting duct epithelium, and oxalate was added to the basolateral side to measure transmembrane transport to the apical side (Figure 6A). UCB treatment increased oxalate concentration on the apical side compared to solvent-treated controls (Figure 6B). This increase was inversely correlated with basolateral oxalate concentrations, indicating enhanced oxalate secretion by UCB (Figure S6A).

Solute carrier family 26 member 6 (*Slc26a6*), a key oxalate transporter facilitating oxalate movement from the basolateral to the apical membrane, was upregulated at both mRNA and protein levels following UCB treatment in MDCK cells (Figure S6B and S6C). In 3D-cultured MDCK cysts, UCB not only elevated slc26a6 expression but also promoted its localization near the apical membrane (Figure 6C). Additionally, UCB-induced slc26a6 overexpression was associated with nuclear translocation of protein kinase C delta (PKC-δ), a kinase involved in regulating oxalate transport. Inhibition of PKC-δ translocation by Rottlerin, a small molecular inhibitor of PKC-δ, blocked UCB-induced slc26a6 upregulation, suggesting that UCB enhances slc26a6 expression via PKC-δ activation (Figure 6D).

*In vivo* studies in renal CaOx crystal depositon rats confirmed that UCB intake increased Slc26a6 expression of renal tubular epithelial cells (Figure 6E, S6D), correlating with higher urinary oxalate excretion (Figure S6E). Furthermore, in the *Drosophila* model of renal CaOx crystal deposition, UCB administration upregulated the expression of *prestin*, the slc26a6 homolog in the Malpighian tubules (Figure S6F). These findings suggest that UCB enhances oxalate secretion by upregulating the membrane expression of the oxalate transporter slc26a6 *in vitro* and *in vivo*.

### UCB promotes CaOx crystal crystallization and aggregation

**Figure 7.**
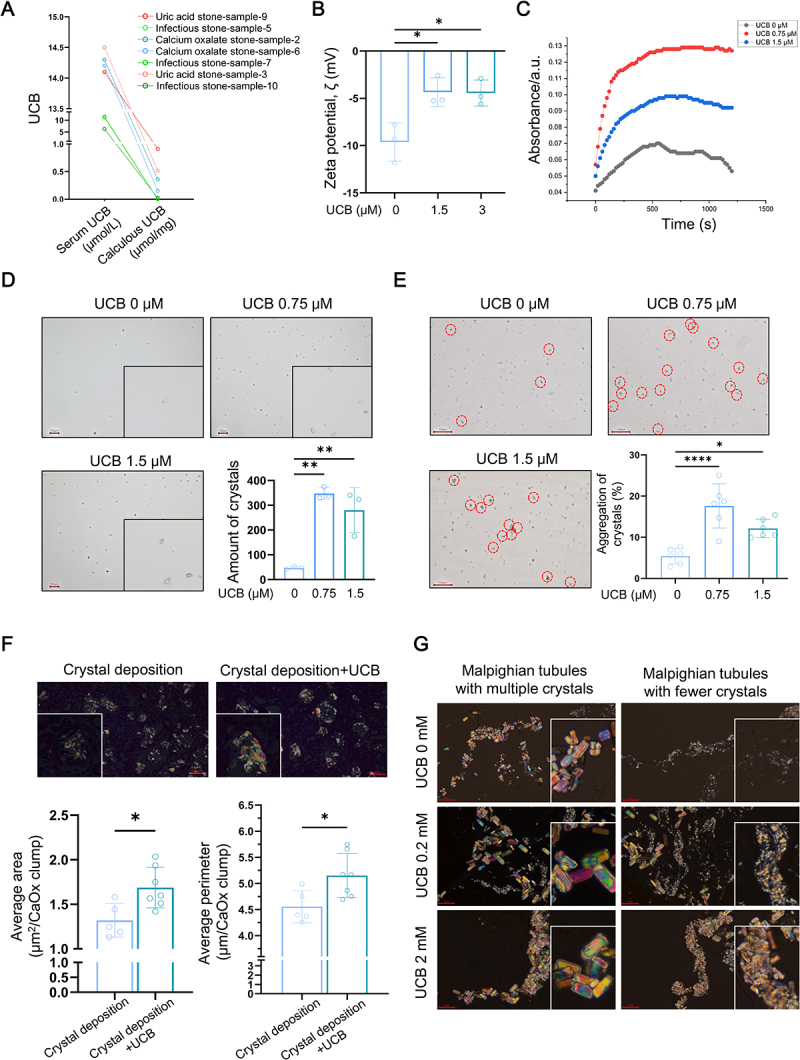
UCB promotes CaOx crystal crystallization and aggregation. (A) The serum UCB and calculous UCB of patients with different kinds of stones were evaluated using automated biochemical analysis and detection kits, respectively. (B) CaOx crystals were prepared with indicate concentrations of UCB. The zeta potential ζ was detected using malvern zetasozer instrument (quantitative data shown are mean with SD, *n* = 3 of each group). (C) CaOx crystals were prepared with indicate concentrations of UCB. The rates of CaOx crystal nucleation were studied by measuring the change of optical density (OD) with time with Cary 500 UV–vis spectrophotometer. (D) CaOx crystals were prepared with indicate concentrations of UCB. The morphology and amounts of formed crystals were analyzed using microscope and software WZCamera (bar = 100 μm; quantitative data shown are mean with SD, *n* = 3 of each group). (E) CaOx crystals were prepared with indicate concentrations of UCB under conditions of shaking. The aggregation of crystals was analyzed using microscope and software WZCamera (bar = 100 μm; quantitative data shown are mean with SD, *n* = 6 of each group). (F) The renal CaOx crystal deposition of rat was identified using polarizing microscope. The average area and perimeter of CaOx clumps were evaluated using software WZCamera (bar = 100 μm; quantitative data shown are mean with SD, *n* = 5 of each group). (G) The CaOx crystal deposition in Malpighian tubules of hyper-Ox diet fly with/without indicate concentration of UCB were evaluated using polarizing microscope (bar = 100 μm). *p < 0.05, **p < 0.01, ***p < 0.0001.

The formation and aggregation of CaOx crystals are critical early determinants of renal CaOx crystal deposition. Therefore, we investigated whether UCB influences these processes. UCB was detected in both CaOx and uric acid stones, suggesting its potential involvement in crystal formation (Figure 7A). To assess this, we measured the impact of UCB on CaOx crystal nucleation and aggregation.

UCB treatment reduced the absolute value of the zeta potential on the crystal surface, indicating enhanced crystal stability (Figure 7B). UCB also promoted the nucleation of CaOx crystals (Figure 7C), increased the formation of COM crystals (Figure 7D), and enhanced crystal aggregation *in vitro* (Figure 7E).

*In vivo*, UCB intake markedly increased the size of renal CaOx crystal deposits in rats (Figure 7F). In the *Drosophila* model, differential CaOx crystal deposition was observed between the two pairs of Malpighian tubules (Figure S7). UCB intake resulted in larger crystals in both pairs, regardless of the baseline crystal deposition (Figure 7G).

Collectively, these findings suggest that UCB contributes to renal crystal deposition by facilitating the crystallization and aggregation of CaOx crystals.

### Relationship between gut microbiota and bilirubin metabolism in renal CaOx crystal deposition rats

Intestinal bilirubin metabolism conducted by gut microbiota is critical for host health. Two key microbial enzymes involved in this process are β-glucuronidase (β-GD) and bilirubin reductase (BilR). CB, along with bile acids and other organic compounds, is secreted into the intestine, where microbial β-GD catalyzes its conversion to UCB. Intestinal UCB can be reabsorbed into the enterohepatic circulation, increasing serum UCB levels, or further metabolized by microbial BilR into stercobilinogen, stercobilin and urobilin, which are excreted in stool and urine. To investigate the relationship between gut microbiota and bilirubin metabolism in renal CaOx crystal deposition hosts, we analyzed gut microbial β-GD-related genes and BilR-related genes in control rats, HYP-induced crystal deposition rats and healthy FMT administered crystal deposition rats.

Microbial metagenome data annotated using the eggNOG database showed an increased relative abundance of β-GD-related unigenes in crystal deposition rats, which was reversed by healthy FMT (Figure S8A). Additionally, a positive correlation was observed between the relative abundance of β-GD-related unigenes and both fecal UCB levels and renal crystal deposition (Figure S8B and S8C).

**Figure 8.**
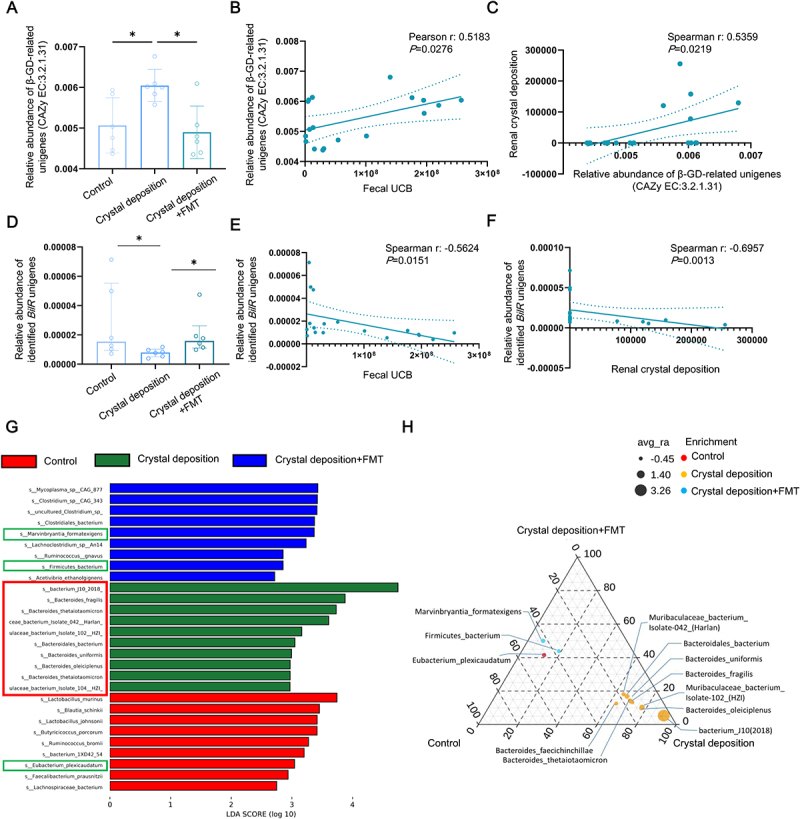
The alteration of gut microbial β-GD related genes, BilR-related genes and related species in renal CaOx crystal deposition rats. (A-C) The β-GD related genes were annotated using CAZy database. The bar chart showing the relative abundance of gut microbial β-GD related unigenes (annotated as EC: 3.2.1.31) between indicated groups (quantitative data shown are median with interquartile range, *n* = 6 of each group) (A); the correlation analysis between the fecal UCB and the β-GD related unigenes (*n* = 18) (B); the correlation analysis between the β-GD related unigenes and the renal crystal deposition (*n* = 18) (C). (D) The bar chart showing the relative abundance of gut microbial BilR unigenes between indicated groups (quantitative data shown are median with interquartile range, *n* = 6 of each group). (E) The correlation analysis between the fecal UCB and the *BilR* unigenes (*n* = 18). (F) The correlation analysis between the *BilR* unigenes and the renal crystal deposition (*n* = 18). (G) Distribution bar chart showing differentially abundant species between the indicated groups identified by LEfSe analysis. β-GD related unigenes derived from eggNOG annotation or from CAZy annotation were employed to annotate β-GD related microbial species using NR database, respectively. Of the species that were significantly upregulated in the crystal deposition group and were significantly recovered in the crystal deposition+FMT group, there were 9 intersecting species (red frame). *BilR* unigenes were employed to annotate BilR related microbial species using NR database, and there are three species that showed changes consistent with changes in their *BilR* unigene abundance respectively (Green frames) (*n* = 6 of each group). (H) species enrichment ternary plots showing differentially abundant species between the indicated group. Differential analysis was performed among the three groups to find species with significant differences. Then, pairwise multiple comparisons were conducted for the significantly different species within the three comparison groups to further determine if the species have significant differences between the pairwise comparison groups (*n* = 6 of each group). *p < 0.05.

The CAZy database is dedicated to the display and analysis of genomic, structural, and biochemical information on carbohydrate-active enzymes, providing classification and related information on enzymes involved in the synthesis, metabolism, and transport of carbon compounds.^32^ The intestinal conversion of CB to UCB is a carbohydrate-active catalytic process, therefore further annotation was carried out using CAZy database and β-GD-related unigenes were taken according to EC: 3.2.1.31, the specific EC number for β-GD activity. Compared to the controls, the relative abundance of β-GD-related unigenes in crystal deposition rats was significantly increased, which was also reversed by healthy FMT (Figure 8A). Additionally, a positive correlation was observed between the relative abundance of β-GD-related unigenes and both fecal UCB levels and renal crystal deposition (Figure 8B,C). Further microbial species information was obtained by annotation of eggNOG-derived and CAZy-derived β-GD-related unigenes using NR database. Results showed that there were nine species were annotated according to eggNOG-derived unigenes and 19 species annotated according to CAZy-derived unigenes, which were significantly upregulated in the Crystal deposition group compared to the Control group, and significantly downregulated in the Crystal deposition + FMT group compared to the Crystal deposition group (Figure S8D). Of these, the common species, such as *Bacterium J10 (2018)*, *Bacteroides_fragilis, Bacteroides_thetaiotaomicron, Muribaculaceae bacterium Isolate-042 (Harlan)*, *Muribaculaceae bacterium Isolate-102 (HZI) etc*., contributed significantly to the relative abundance alteration of β-GD-related unigenes (Figure 8G,H, S9A). The result of enzyme activity detection showed that the activity of β-GD in crystal deposition rats was significantly increased, which was also reversed by healthy FMT (Figure S9C).

In contrast, the relative abundance of unigenes homologous with *BilR* was significantly decreased in crystal deposition rats, and this reduction was reversed by healthy FMT (Figure 8D). A significant reverse correlation was observed between the relative abundance of *BilR* unigenes and both fecal UCB levels and renal crystal deposition (Figure 8E,F). The decrease in *BilR* unigenes was mainly attributed to changes in species *Marvinbryantia formatexigens*, *Eubacterium plexicaudatum* and *Firmicutes bacterium* (Figure 8G,H, S9B). To investigate the expression level of *BilR*, we used qRT-PCR to test the representative *BilR* mRNA. We observed that comparing with the Control or the Crystal deposition + FMT group, the Ct values of *BilR* mRNA were larger in the Crystal deposition group (Figure S9D-H), suggesting that the transcriptional expressions of *BilR* by gut microbiota are less active in renal CaOx crystal deposition rats.

There are eight species which were also identified as BilR-related species in a fecal sample from healthy human (Figure S10A), of which *Faecalibacterium prausnitzii* and *Gemmiger formicilis* are the top 20 most abundant species in healthy human (Figure S10B), and the correlation heat map shown the significant correlation between their abundances and fecal UCB, fecal Stercobilin, renal crystals in rats model, respectively (Figure S10C), suggesting potential of *Faecalibacterium prausnitzii* and *Gemmiger formicilis* application for intestinal UCB metabolism and renal CaOx crystal deposition prevention.

**Figure 9.**
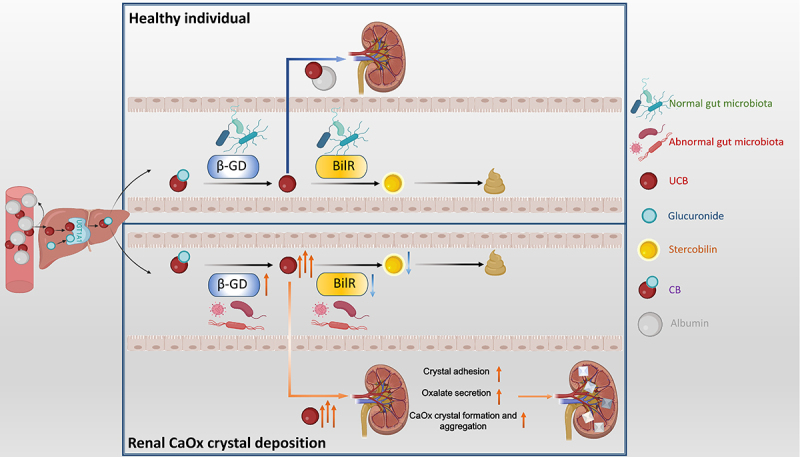
The schematic diagram of gut microbiota-regulated UCB metabolism driving renal CaOx crystal deposition.

These findings demonstrate that gut microbiota involved in intestinal bilirubin metabolism are dysregulated in rats with renal CaOx crystals. This dysbiosis may be linked to altered serum UCB levels and the formation of renal CaOx crystal deposition (Figure 9).

## Discussion

Gut microbiota are vital for the maintenance of metabolic homeostasis in the host. The various enzymes expressed by gut microbiota catalyze the intestinal conversion of both foodborne and endogenous metabolites. These, in turn, play a crucial role in the physiology and disease process of the host. In this study, we demonstrated that UCB, a gut microbial β-GD and BilR-regulated metabolite, plays a vital role in renal CaOx crystal deposition, which is one of the primary initiating factors of CaOx formation. We found that the serum UCB/Alb ratio is an independent risk factor for CaOx stone formation, additionally, UCB promotes renal tubular epithelial cell-crystal adhesion, enhances urinary oxalate excretion, and facilitates crystal formation and aggregation, leading to renal CaOx crystal depositon. To our knowledge, this is the first study identifying UCB activity as a promoter of renal CaOx crystal deposition.

Traditionally, UCB was considered a metabolic waste product with neonatal neurotoxicity. However, recent research has highlighted bilirubin’s regulatory role in various biological processes, acting as a “yellow hormone” at physiological concentrations.^33^ UCB’s lipophilicity and free availability enable it to cross cell membranes, although most UCB is bound to albumin in the blood, which restricts its cellular uptake.^34^ Certain conditions, such as hypoxia or specific pharmacological agents, can disrupt UCB-Alb binding, leading to increased free UCB levels and enhanced cytotoxicity.^35–37^ In this study, UCB activity on renal tubular epithelial cells was completely inhibited by 10% FBS, indicating the protective effect of Alb. Additionally, UCB is converts to CB in hepatocytes,^34^ reducing UCB’s transmembrane activity. Therefore, the UCB/Alb ratio is a more accurate indicator of UCB activity *in vivo* compared to total bilirubin or DBIL.^38,39^

Bilirubin is known for its potent antioxidant properties,^40^ and mice lacking bilirubin are highly susceptible to oxidative stress.^41^ Mild hyperbilirubinemia is associated with lower adiposity, decreased metabolic syndrome risk, and reduced prevalence of nonalcoholic fatty liver disease,^42^ diabetes,^43,44^ and certain cancers.^43^ Individuals with Gilbert’s syndrome, characterized by chronic mild hyperbilirubinemia due to *UGT1A1* mutations, have lower incidences of cardiovascular disease, diabetes, and all-cause mortality.^45–47^ These findings demonstrate the protective effects of mild hyperbilirubinemia.

Oxidative stress plays a critical role in renal CaOx crystal deposition, and UCB’s antioxidant capacity may contribute to this process. In this study, both CaOx stone patients and crystal deposition rats exhibited higher serum UCB activity, leading us to hypothesize that the higher UCB activity may be a compensatory response to oxidative stress. However, further findings revealed that UCB significantly enhanced renal CaOx crystal deposition in both rat and *Drosophila* models. In particular, UCB even induced more CaOx crystal deposition in normal fed flies, indicating UCB is a risk factor for renal CaOx crystal deposition.

Renal tubular epithelial cell apoptosis also contributes to renal CaOx crystal deposition. Apoptotic cells are more likely to adhere urinary crystals, promoting crystal aggregation.^48^ Even low concentrations of UCB can trigger apoptosis in various cell types.^38,49–51^ Our study confirmed that UCB induces significant apoptosis in renal tubular epithelial cells *in vitro* and enhances CaOx crystal adhesion.

Urinary oxalate is another important risk factor for renal CaOx crystal deposition and related CaOx stone formation. Urinary oxalate originates from glomerular filtration and renal tubular secretion, the latter regulated by oxalate transporters, such as Slc26a6.^52^ We demonstrated that UCB significantly increases oxalate secretion by upregulating renal Slc26a6 expression in crystal deposition rats. In *Drosophila*, UCB also upregulated *prestin* expression in Malpighian tubules, the homologous gene to Slc26a6, confirming UCB’s role in promoting renal tubular oxalate secretion.

We also discovered that UCB enhances CaOx crystal formation and aggregation. UCB’s strong binding affinity to calcium ions may facilitate CaOx nucleation, while reducing the negative charge on the crystal surface may promote aggregation. In summary, while UCB possesses strong antioxidant properties, it paradoxically increases the risk of renal CaOx crystal deposition by promoting renal tubular apoptosis, enhancing oxalate secretion, and facilitating crystal formation and aggregation.

The gut microbiota plays a critical role in UCB metabolism.^53–55^ Microbial enzymes, such as β-GD and BilR, regulate the source, the enteral excretion and reabsorption of intestinal UCB.^11,27,56^ Disruptions in gut microbiota can alter serum UCB levels.^57^ We identified significant dysbiosis in the gut microbiota of renal CaOx crystal deposition rats, characterized by altered abundances of β-GD- and BilR-related genes and species. We propose that this dysbiosis elevates serum UCB levels by modulating intestinal UCB metabolism. *Faecalibacterium prausnitzii* and *Gemmiger formicilis* were identified as intestinal BilR-related species of healthy human in this study. Both of *Faecalibacterium prausnitzii* and *Gemmiger formicilis* are depleted in IBD cohorts,^58^ and biliary bilirubin and the incidence of gallstones are increased in inflammatory bowel disease (IBD) patients with ileal disease,^59^ highlighting the potential association of these species and bilirubin metabolism. We found that *Faecalibacterium prausnitzii* and *Gemmiger formicilis* were the major species in healthy human feces, and they were positively correlated with both fecal UCB levels and renal crystal deposition in rats. These findings suggest that the cocktail of *Faecalibacterium prausnitzii* and *Gemmiger formicilis* may be safe and potential for reducing UCB and preventing renal CaOx crystal deposition.

A variety of gut microbiota-derived metabolites participate in the regulation of intestinal and kidney health, as well as renal CaOx crystal deposition and related CaOx stone formation. SCFAs is a major class of metabolites derived from fermentative catalysis of microbiota, serving as energy sources for enterocytes. They can maintain gut structural barriers and immunmodulation,^60^ in turn, reduce renal CaOx crystal deposition.^9,61^ Bile acids (BAs) play a pivotal role in the process of digesting and absorbing fats and fat-soluble vitamins within the intestinal tract. In addition to this primary function, they also serve as signaling molecules that can regulate a multitude of biological processes, including immune and inflammatory responses.^62,63^ Changes in the content and composition of human BAs can modulate renal ferroptosis related-CaOx crystal deposition.^14^ In addition, oral conjugated-BA has been demonstrated to reduce urinary oxalate excretion and CaOx nephrolithiasis in a patient with short bowel syndrome.^64^ The extant research does not provide evidences for direct biochemical reactions between bilirubin, SCFAs and BAs, however, indirect metabolic cross-talk in the intestinal environment is a possibility, and this could be mediated, for example, by the gut microbiota. BAs affects the abundance, diversity and metabolic activity of microbiota.^65^ Microbiota expressed β-GD not only catalyzes intestinal bioconversion of bilirubins, but also hydrolyzes BAs-glucuronic conjugates and overproduces most BAs in the serum and liver.^66^ The ingestion of bilirubin has been demonstrated to induce a significant enrichment of microbial secondary bile acid metabolism in mice with lung adenocarcinoma.^67^ Butyrate (a typical SCFA) upregulates the expression of tight junction proteins in intestinal epithelial cells and promotes mucus secretion from goblet cells,^68^ which enhances the intestinal physical barrier function and may limit excessive intestinal bilirubin absorption into the enterohepatic circulation. Our previous study also found that mature vinegar enriched with acetic acid (a typical SCFA) was able to significantly restore disordered gut microbiota and urinary bilirubin of EG-induced renal CaOx deposition rats.^69^ The above inspires the mutually modulated relationships between SCFAs, BAs and bilirubin metabolism. Therefore, it is worthwhile to further explore their communication in the development of renal CaOx crystal deposition.

However, some limitations in this study should be noticed. The development of mature CaOx stone formation is a complex multistep and chronic process. It involves several stages, including intratubular initial precipitation, adhesion and deposition of urinary CaOx crystals, renal injury, formation of Randall plaque and collecting ducts Randall plug, as well as urinary crystals adhering to Randall plaque, to form mature and clinical stone.^70^ In this study, despite that significant CaOx crystal deposition could be observed, no signs of mature stone formation were evident in any of the cells, rats or Drosophila models employed. Additionally, although Drosophila Malpighian tubules generate a primary urine by transcellular transport, serving a comparable excretory function and sharing conserved ion transport mechanisms involved in crystal formation,^71^ Malpighian tubules are not true anatomical analogs of human kidneys. Therefore, the present study provides limited evidence for the regulatory role of gut microbiota-related UCB metabolism on renal CaOx crystalline materials. Furthermore, additional research is necessary in mature stone-bearing models and in clinical settings, in order to determine the efficacy of interventions targeting gut microbiota and UCB activity for the prevention of CaOx stone.

## Conclusion

We identify gut microbiota-associated UCB activity as a key factor promoting renal CaOx crystal deposition. Modulating intestinal bilirubin metabolism or inhibiting UCB activity may represent an effective strategy for preventing renal CaOx crystal deposition and CaOx nephropathies.

## Supplementary Material

Supplementary materials R-clean for typesetting.docx

## Data statement

All data analyzed are included in this published article and its supplementary information files. Datasets not directly provided in the publication are available from the corresponding author upon reasonable request as stated in the Data Availability Statement.
