# Supplementary material for: Gut microbiota-regulated unconjugated bilirubin metabolism drives renal calcium oxalate crystal deposition
Source: Gut Microbes. 2025 Aug 24;17(1):2546158. doi: 10.1080/19490976.2025.2546158 (PMC12377128; doi:10.1080/19490976.2025.2546158)
Supplement: Supplementary materials R-clean for typesetting.docx [file KGMI_A_2546158_SM0303.docx]

Supplementary Materials

**Gut microbiota-regulated unconjugated bilirubin metabolism drives** **renal calcium oxalate crystal deposition**

Shujue Li^2,5#^, Wenzheng Wu^1,#^, Yuhao Zhou^1,#^, Shike Zhang^1,#^, Daqiang Wei^1^, Mingzhao Zhu^1^, Xiaoling Ying^1^, Xinyuan Sun^2^, Hongxing Liu^2^, Wei Zhu^2^, Daolin Tang^3^, Renjie Jiao^1,4^, Guohua Zeng^2^, Xiaolu Duan^2,*^, Jinbao Liu^1,5,*^, and Wenqi Wu^1,*^

^1^ Department of Urology, the Second Affiliated Hospital, Guangzhou Medical University, Guangzhou 510260, China

^2^ Department of Urology, The First Affiliated Hospital, Guangdong Provincial Key Laboratory of Urological Diseases, Guangdong Engineering Research Center of Urinary Minimally Invasive Surgery, Robot and Intelligent Equipment, Guangzhou Institute of Urology, Guangzhou Medical University, Guangzhou 510230, China

^3^ Department of Surgery, UT Southwestern Medical Center, Dallas, Texas 75390, USA

^4^ Sino-French Hoffmann Institute, Guangzhou Medical University, Guangzhou, 511436, China

^5^ Affiliated Cancer Hospital & Institute of Guangzhou Medical University, Guangzhou Municipal and Guangdong Provincial Key Laboratory of Protein Modification and Disease, State Key Laboratory of Respiratory Disease, School of Basic Medical Sciences, Guangzhou Medical University, Guangzhou 511436, China

^#^ Contributed equally.

*Corresponding authors:

Wenqi Wu, Jinbao Liu and Xiaolu Duan

E-mail: 2009681012@gzhmu.edu.cn; jliu@gzhmu.edu.cn; 94302304@qq.com;

**Contents**

Supplementary Figure S1**……………………………………………………………3**

Supplementary Figure S2**……………………………………………………………4**

Supplementary Figure S3**……………………………………………………………5**

Supplementary Figure S4**……………………………………………………………6**

Supplementary Figure S5**……………………………………………………………7**

Supplementary Figure S6**……………………………………………………………8**

Supplementary Figure S7**……………………………………………………………10**

Supplementary Figure S8**……………………………………………………………11**

Supplementary Figure S9**……………………………………………………………12**

Supplementary Figure S10**……………………………………………………………14**

Supplementary Table S1**……………………………………………………………15**

Supplementary Table S2**……………………………………………………………16**

Supplementary Table S3**……………………………………………………………18**

Supplementary Table S4**……………………………………………………………19**

**Supplementary Figure S1**

**
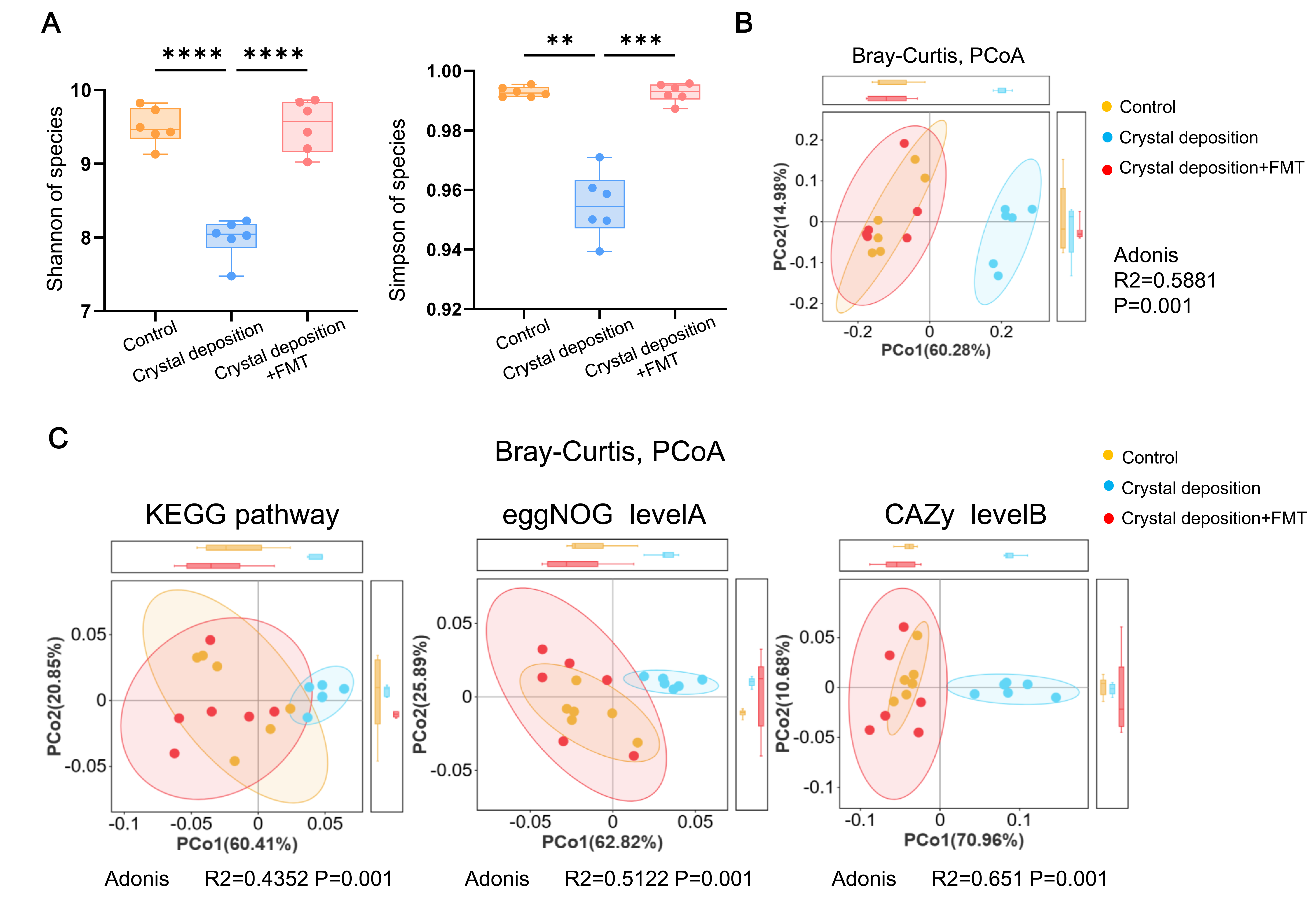
**

**Supplementary Figure S1**. **Dysbiosis of the gut microbiota in renal CaOx crystal deposition rats and healthy-FMT induced recovery of the gut microbial community**

(**A**) Comparison of alpha diversity at special level between indicated groups, including Shannon and Simpson-index (Quantitative data shown are median with interquartile range, n=6 of each group). (**B**) The beta diversity of gut microbial species showed by PCoA based on Bray-Curtis distance. (**C**) The beta diversity of gut microbial function showed by PCoA based on Bray-Curtis distance. **P <0.01, ***P <0.001, ****P <0.0001.

**Supplementary Figure S2**


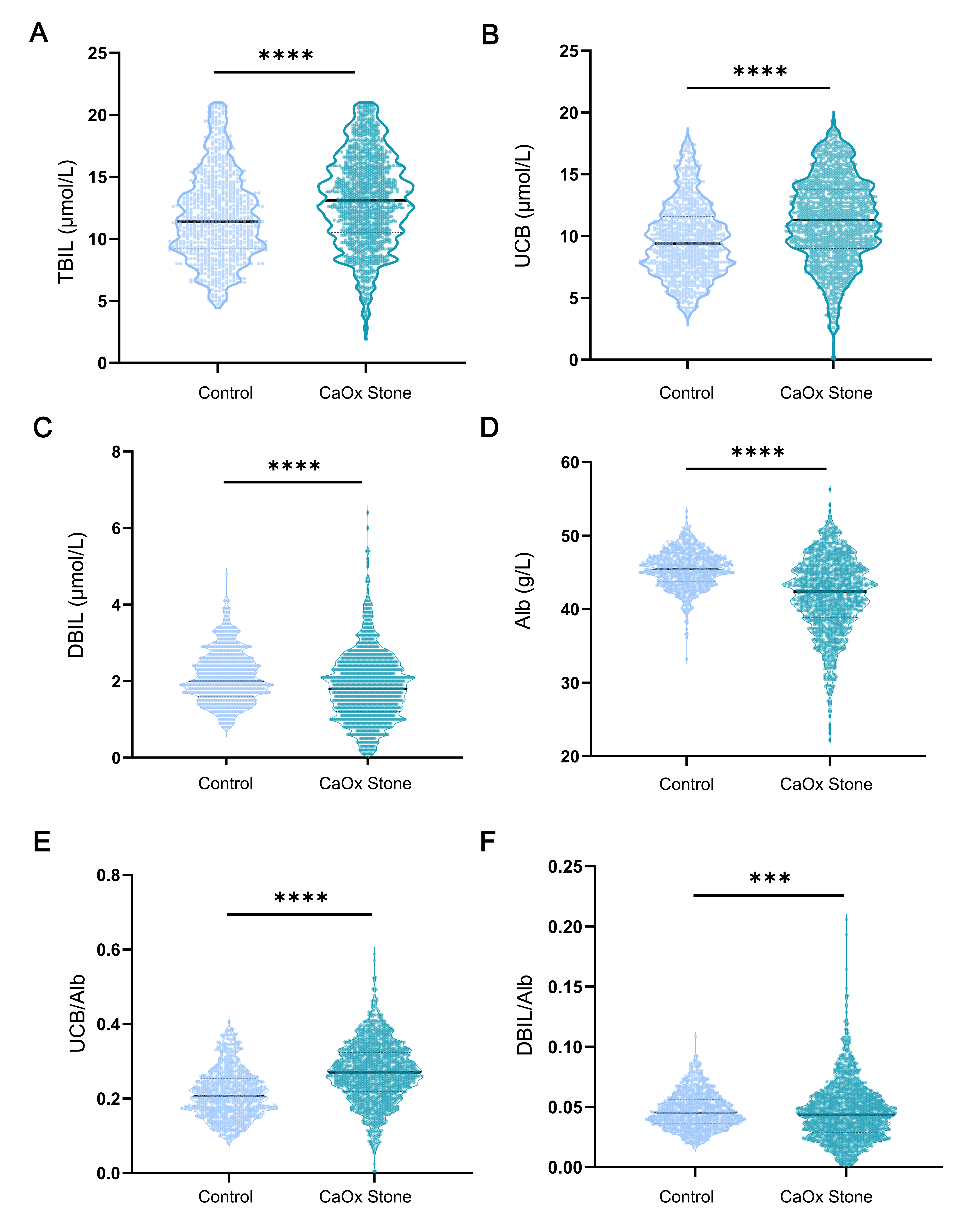


**Supplementary Figure S2**. **The comparison of circulatory UCB activity-relative laboratory indexes between the control group and the CaOx-stone group before matching of gender and age.**

(**A**-**D**) The levels of serum total bilirubin (A), UCB (B), DBIL (C) and Alb (D) were evaluated using standard chemical methods. (**E** and **F**) The levels of UCB/Alb ratio (E) and DBIL/Alb ratio (F) were calculated. Quantitative data shown are median with interquartile range. n=822 of control group, n=1334 of CaOx-urolithiasis group. *** P <0.001, ****P <0.0001.

**Supplementary Figure S3**


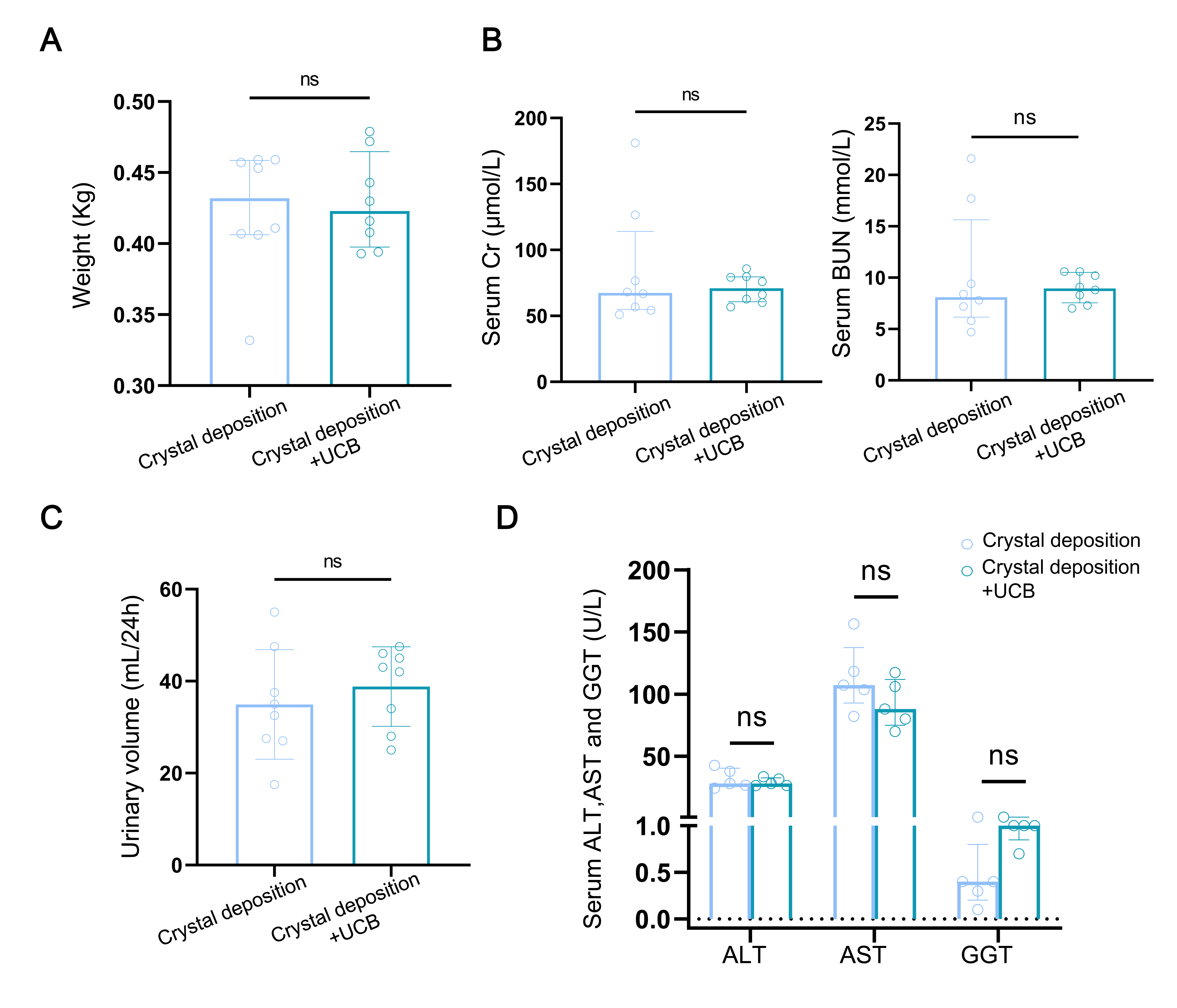


**Supplementary Figure S3**. **The weight, 24h urine volume, renal function, and liver function of rats in the Crystal deposition group and the Crystal deposition + UCB group**

(**A**) The weight of rats. (**B**) The serum indicators of renal function: Cr and BUN. (**C**) The urinary volume of rats (24h). (**D**) The serum indicators of liver function: ALT, AST and GGT. Quantitative data shown are mean with SD, n=8 of each group, ns. no significant different.

**Supplementary Figure S4**


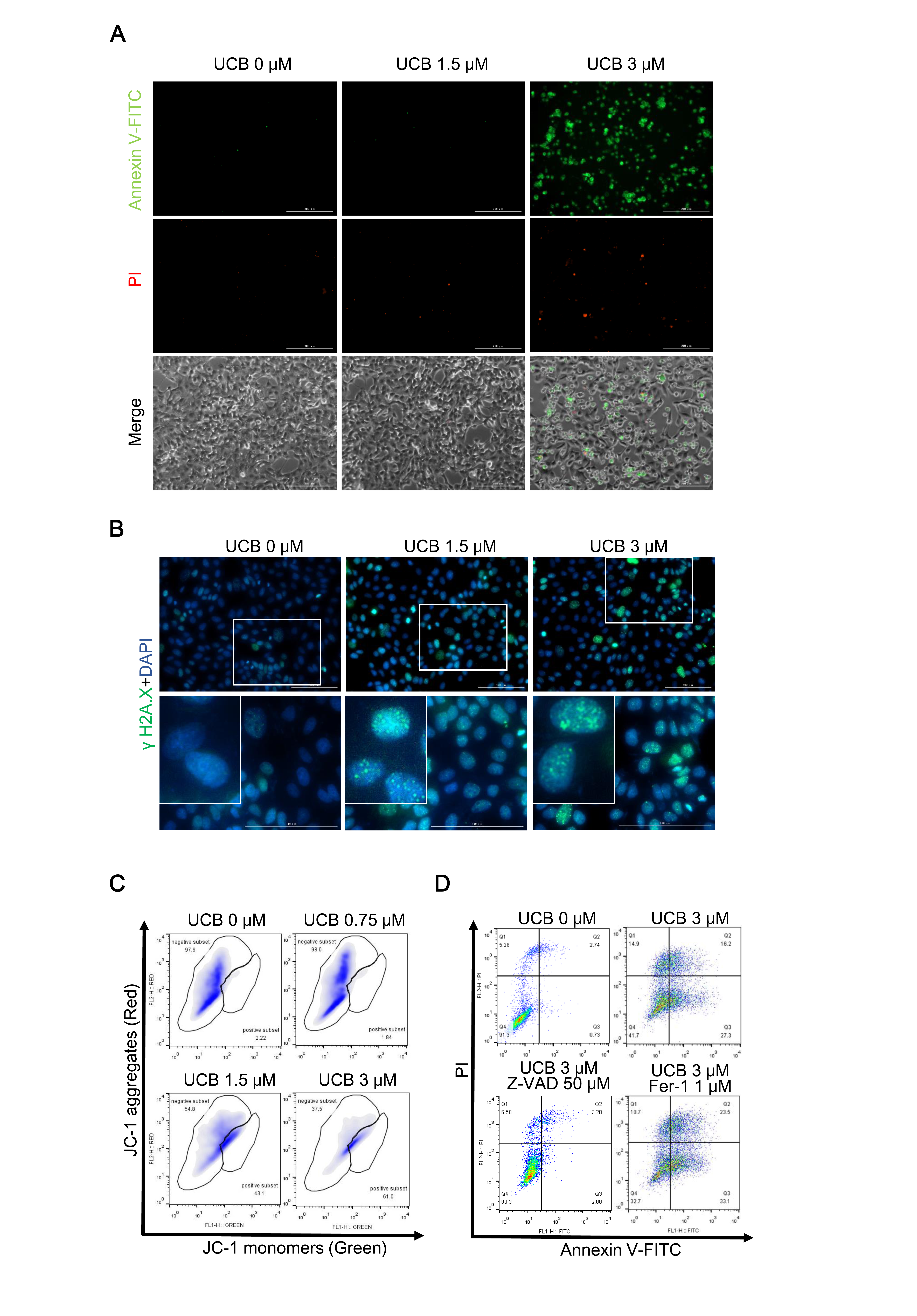


**Supplementary Figure S4**. **UCB induces DNA damage, mitochondrial damage and apoptosis of MDCK cells**

(**A**) MDCK cells were treated with UCB in 1% FBS environment for 10 hours. Cell death was detected using Annexin V-FITC and PI staining (Bar: 200 μm). (**B**-**C**) MDCK cells were treated with UCB in 1% FBS environment for 8 hours. DNA damage indicated by γH2AX foci were determined by immunofluorescence (B) (Top layer Bar: 200 μm; Bottom layer Bar: 100 μm). Mitochondrial damage indicated by mitochondrial Δψm alteration was assessed by flowcytometry after stained with 5, 5′, 6, 6′-tetrachloro-1,1′,3,3′-tetraethylbenzimidazolcarbocyanine iodide (JC-1) (C). (**D**) MDCK cells were treated with UCB in 1% FBS environment for 24 hours. Cell death was detected using Annexin V-FITC-PI staining and flow cytometry.

**Supplementary Figure S5**

**Supplementary Figure S5**. **UCB induces tight junction disruption of MDCK cell layer**

(**A**-**B**) MDCK cells were treated with UCB and Z-VAD in 1% FBS environment for 10h. Expression of tight junction proteins were detected using western blotting (A) and immunofluorescence (B) (Bar: 200 μm).

**Supplementary Figure S6**


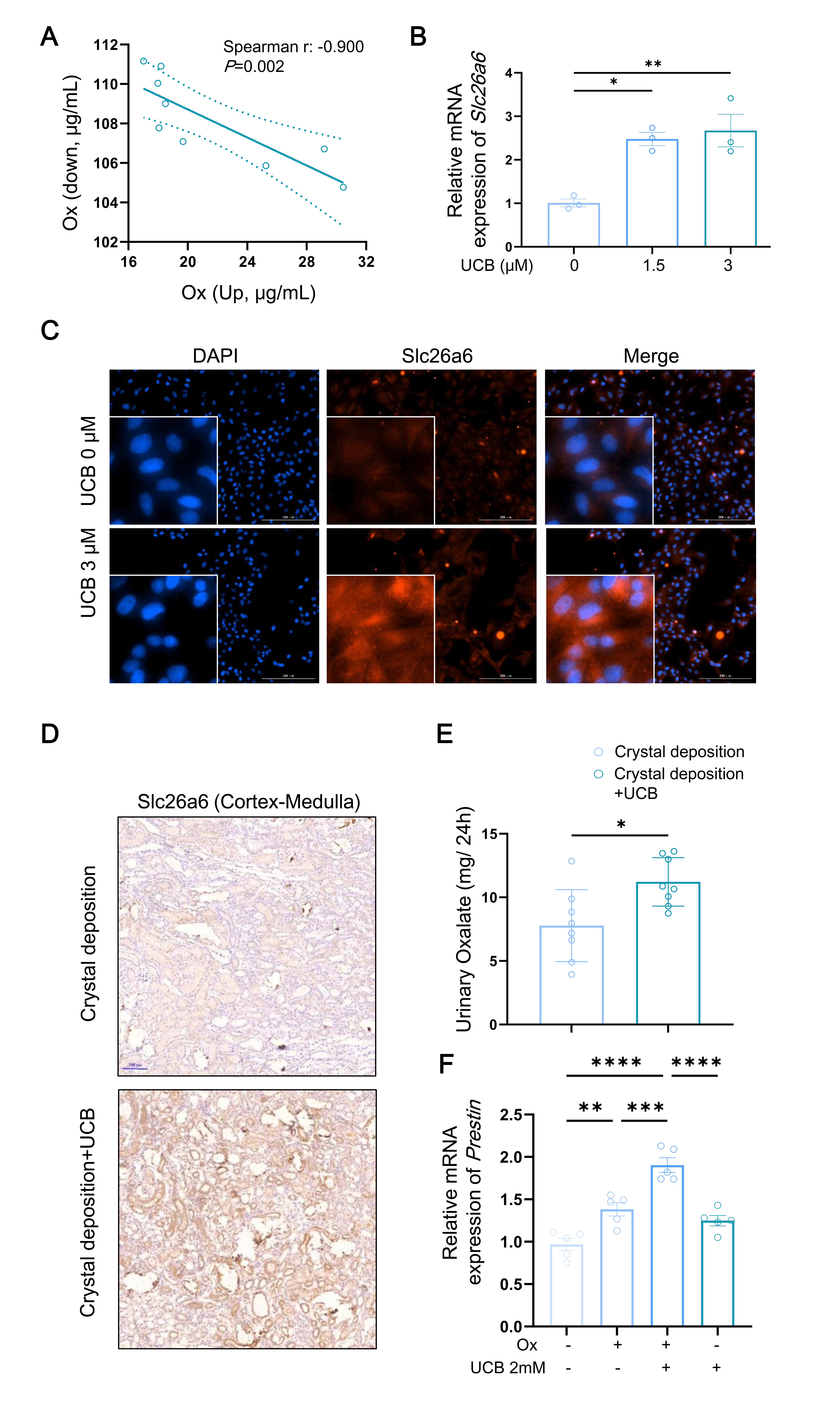


**Supplementary Figure S6**. **UCB promotes the expression of Slc26a6 in renal tubular epithelial and increases urinary oxalate**

(**A**) In the *in vitro* cell model that simulates renal tubular epithelium, MDCK cells were treated with UCB in 1% FBS environment for 10 hours, followed by oxalate treatment in basal membrane side for 2 hours. The oxalate concentrations of upper medium and lower medium were detected using ion exchange chromatography. The oxalate concentration of upper medium was negatively correlated with lower chamber, n=9). (**B**) MDCK cells were treated with UCB in 1% FBS environment for 24 hours. The alteration of Slc26a6 mRNA expression was analyzed using RT-PCR (Quantitative data shown are mean with SEM, n=3 of each group). (**C**) 2D-cultured MDCK cells were treated with UCB in 1% FBS environment for 10 hours. The expression of Slc26a6 was detected using immunofluorescence. (Bar: 200 μm). (**D**) The renal tubular Slc26a6 expression of rats in the Crystal deposition group and the Crystal deposition + UCB group were evaluated using IHC analysis (Bar: 100 μm). (**E**) The urinary oxalate of rats in the Crystal deposition group and the Crystal deposition + UCB group were detected using ion chromatography (Quantitative data shown are mean with SD, n=8 of each group). (**F**) The relative mRNA expression of *Prestin*, the homologous gene of slc26a6, in the Malpighian tubules of flies with indicate treatments were analyzed using RT-qPCR (Quantitative data shown are mean with SEM, n=5 each group). *P <0.05, **P <0.01, ***P <0.001, ****P <0.0001.

**Supplementary Figure S7**


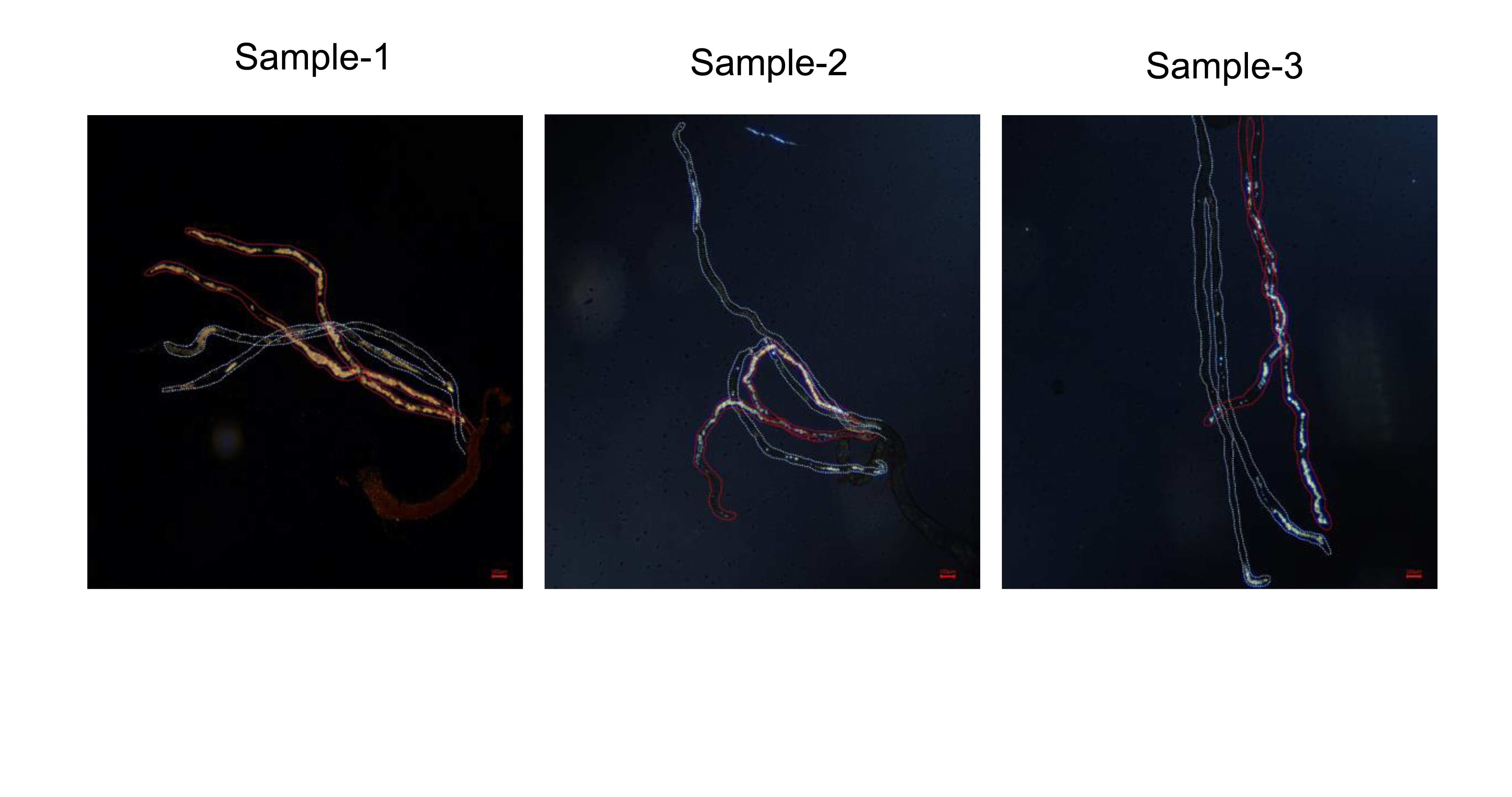


**Supplementary Figure S7. The deposition of CaOx crystals induced by hyperoxalate are different between the two pairs of Malpighian tubules within the same fly.**

Two pairs of Malpighian tubules of one drosophila were delineated using dashed lines of varying colors (Red: Tubules with multiple crystals; Blue: Tubules with fewer crystals).

**Supplementary Figure S8**


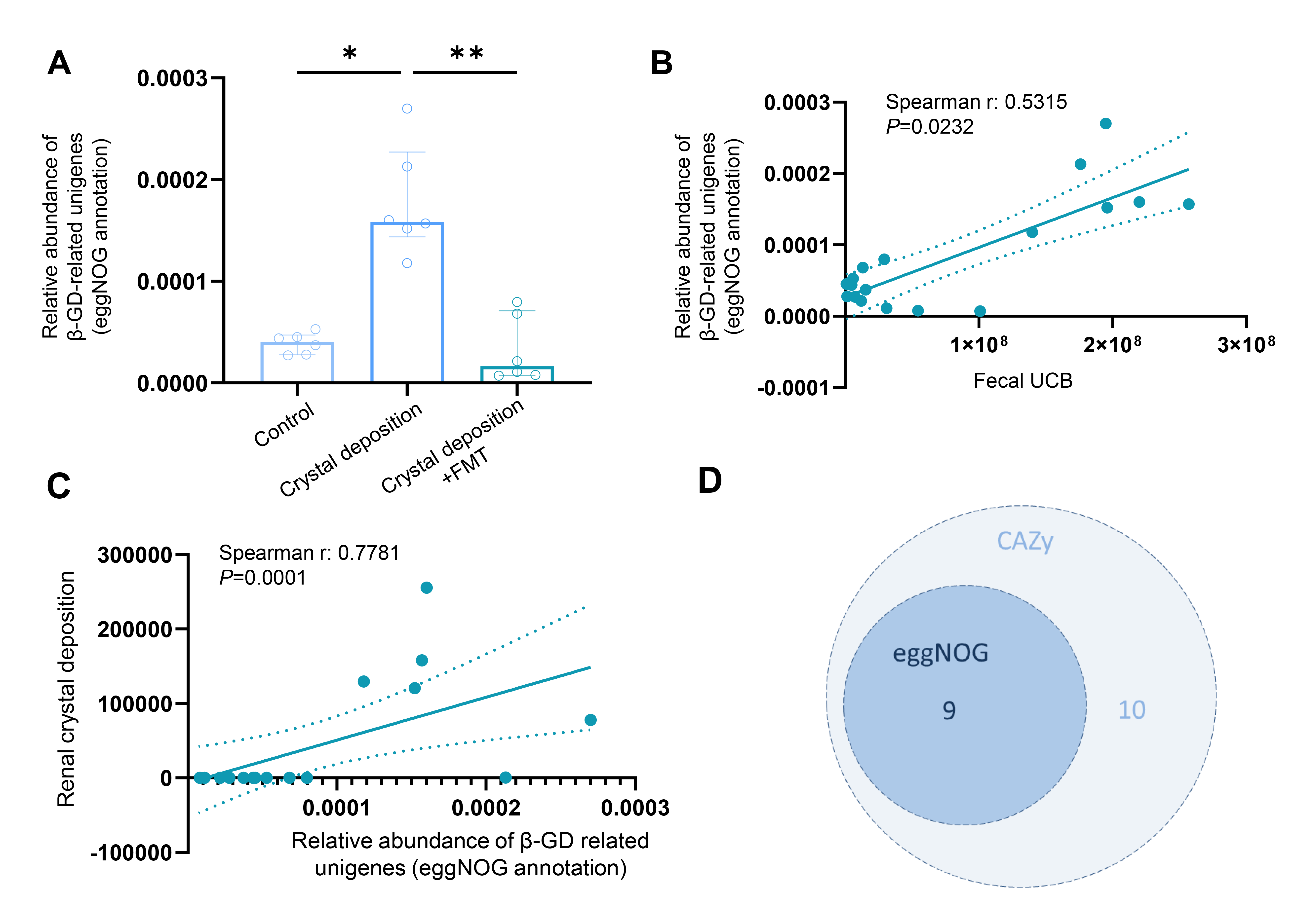


**Supplementary Figure S8. The alteration of gut microbial β-GD related genes and species in renal CaOx crystal deposition rats. Related to Figure 8.**

(**A-C**) The β-GD related unigenes were annotated using eggNOG database. The bar chart showing the relative abundance of gut microbial β-GD related unigenes between indicated groups (Quantitative data shown are median with interquartile range, n=6 of each group) (A); the correlation analysis between the fecal UCB and the β-GD related unigenes (n=18) (B); the correlation analysis between the β-GD related unigenes and the renal crystal deposition (n=18) (C). (**D**) β-GD related unigenes derived from eggNOG annotation or from CAZy annotation were employed to annotate β-GD related microbial species using NR database, respectively. There were 9 species annotated by β-GD related unigenes from eggNOG and 19 species annotated by β-GD related unigenes from CAZy database, which were significantly upregulated in the Crystal deposition group compared to the Control group, and significantly downregulated in the Crystal deposition +FMT group compared to the stone group. The Venn diagram showing the number of eggNOG-CAZy crossover β-GD related microbial species. * P <0.05, **P <0.01.

**Supplementary Figure S9**

**
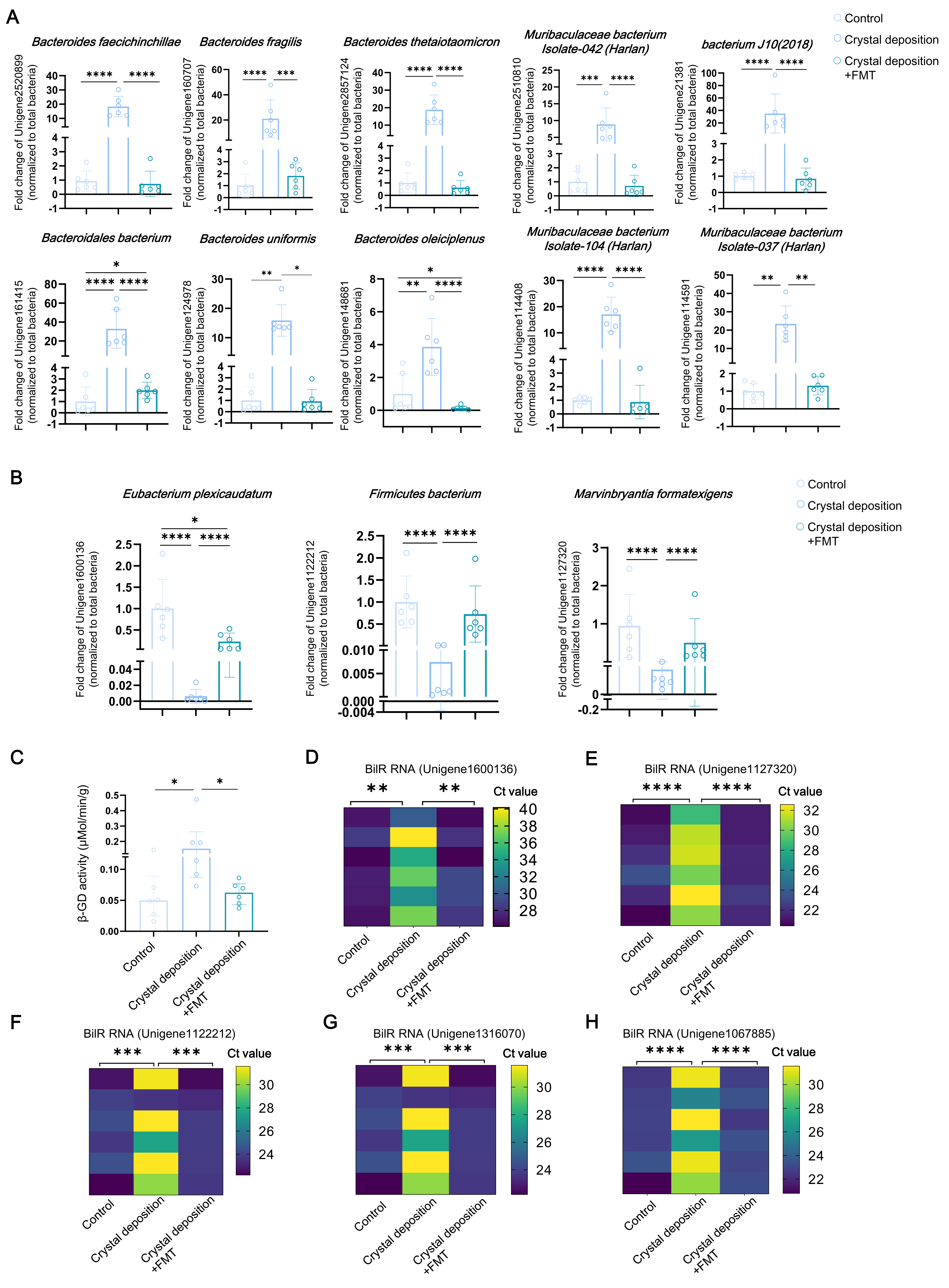
**

**Supplementary Figure S9. Validation of changes in the abundance of representative unigenes/species and in key microbial functions**

(**A**) qPCR analysis of representative β-GD related unigenes/species (Quantitative data shown are mean with SD, n=6 of each group). (**B**) qPCR analysis of representative *BilR* unigenes/species (Quantitative data shown are mean with SD, n=6 of each group). (**C**) Fecal β-GD activity was detected using p-Nitrophenol assay (Quantitative data shown are median with interquartile range, n=6 of each group). (**D-H**) RT-qPCR analysis of mRNA expression of representative *BilR* unigenes and heatmaps shown the Ct values (n=6 of each group). * P <0.05, **P <0.01, ***P <0.001, **** P <0.0001.

**Supplementary Figure S10**


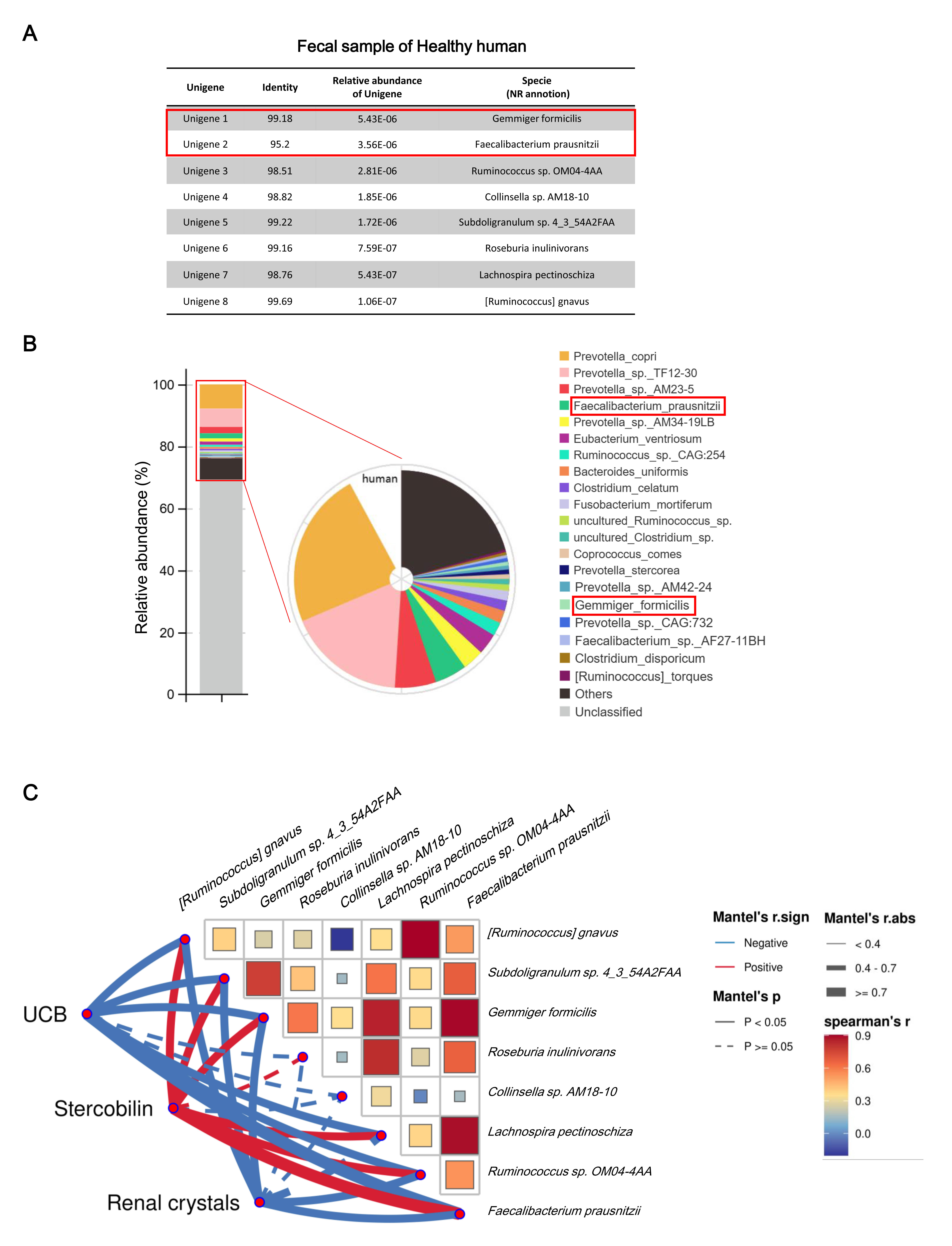


**Supplementary Figure S10. Annotated *BilR* unigenes and related species in fecal microbiota of healthy human**

(**A**) The relative abundance of *BilR* unigenes and annotated species in fecal sample of healthy human (Red picture frame: Top2 unigenes). (**B**) The distribution of microbial species community of the fecal sample of healthy human (Top 20 species; Red picture frame: BilR related species). (**C**) The correlation heat map described the relationship between the identified BilR-related species (common in healthy human and rat) and fecal UCB, fecal Stercobilin, renal crystals in rats (n=18).

**Supplementary Table S1. Gender, Age and laboratory information by the status of CaOx stones before matching of gender and age**

| Variables | Total (n = 2156) | Control  (n = 822) | CaOx-urolithiasis  (n = 1334) | *P* |
| --- | --- | --- | --- | --- |
|  |  |  |  |  |
| Age, M (Q₁, Q₃) | 46.00 (36.00, 55.00) | 38.00 (33.00, 44.75) | 52.00 (42.00, 59.00) | **<.001** |
| Scr (umol/L), M (Q₁, Q₃) | 83.00 (68.60, 102.43) | 69.90 (64.03, 79.97) | 93.55 (78.03, 114.77) | **<.001** |
| ALT (U/L), M (Q₁, Q₃) | 18.40 (13.70, 26.00) | 15.60 (11.60, 22.35) | 20.00 (15.00, 28.00) | **<.001** |
| TP (g/L), M (Q₁, Q₃) | 74.70 (70.70, 77.90) | 76.50 (73.90, 78.90) | 73.10 (68.80, 76.80) | **<.001** |
| TBA (umol/L), M (Q₁, Q₃) | 4.20 (2.90, 6.66) | 4.50 (3.60, 6.18) | 3.91 (2.26, 7.06) | **<.001** |
| GGT (U/L), M (Q₁, Q₃) | 19.00 (13.80, 27.80) | 17.50 (13.60, 26.20) | 19.00 (14.00, 29.00) | **0.016** |
| TC (mmol/L), M (Q₁, Q₃) | 5.17 (4.58, 5.86) | 5.00 (4.49, 5.66) | 5.35 (4.69, 6.08) | **<.001** |
| TG (mmol/L), M (Q₁, Q₃) | 1.29 (0.89, 2.00) | 1.08 (0.79, 1.56) | 1.58 (1.05, 2.56) | **<.001** |
| HDL-C (mmol/L), M (Q₁, Q₃) | 1.36 (1.15, 1.62) | 1.50 (1.30, 1.73) | 1.22 (1.02, 1.42) | **<.001** |
| LDL-C (mmol/L), M (Q₁, Q₃) | 3.17 (2.69, 3.71) | 3.27 (2.86, 3.76) | 3.06 (2.53, 3.67) | **<.001** |
| UA (umol/L), M (Q₁, Q₃) | 366.10 (303.95, 446.00) | 332.50 (275.30, 397.75) | 397.00 (338.00, 476.00) | **<.001** |
| WBC (10^9/L), M (Q₁, Q₃) | 6.59 (5.50, 7.91) | 6.00 (5.10, 7.10) | 6.95 (5.79, 8.46) | **<.001** |
| NEUT# (10^9/L), M (Q₁, Q₃) | 3.90 (3.00, 5.10) | 3.30 (2.70, 4.10) | 4.40 (3.30, 5.80) | **<.001** |
| LYMPH# (10^9/L), M (Q₁, Q₃) | 2.00 (1.60, 2.50) | 2.00 (1.70, 2.40) | 2.00 (1.60, 2.50) | 0.092 |
| MONO# (10^9/L), M (Q₁, Q₃) | 0.40 (0.30, 0.50) | 0.40 (0.40, 0.50) | 0.40 (0.30, 0.50) | **<.001** |
| RBC (10^12/L), M (Q₁, Q₃) | 4.72 (4.36, 5.14) | 4.53 (4.27, 4.87) | 4.86 (4.46, 5.27) | **<.001** |
| Hb (g/L), M (Q₁, Q₃) | 137.00 (126.00, 148.00) | 135.00 (128.00, 144.25) | 138.00 (125.00, 150.00) | **0.015** |
| PLT (10^9/L), M (Q₁, Q₃) | 238.00 (203.00, 280.00) | 243.00 (210.00, 280.00) | 235.50 (199.00, 279.75) | **<.001** |
| Urine pH, M (Q₁, Q₃) | 6.00 (6.00, 6.50) | 6.00 (5.00, 6.50) | 6.00 (6.00, 6.50) | **<.001** |
| Gender, n(%) |  |  |  | **<.001** |
| Male | 1032 (47.87) | 202 (24.57) | 830 (62.22) |  |
| Female | 1124 (52.13) | 620 (75.43) | 504 (37.78) |  |
| KET, n(%) |  |  |  | **0.025** |
| Negative | 1705 (97.82) | 463 (98.93) | 1242 (97.41) |  |
| Trace | 26 (1.49) | 1 (0.21) | 25 (1.96) |  |
| Positive | 12 (0.69) | 4 (0.85) | 8 (0.63) |  |
| SG, n(%) |  |  |  | **0.004** |
| <=1.005 | 310 (17.72) | 96 (20.25) | 214 (16.78) |  |
| 1.005-1.030 | 1363 (77.93) | 369 (77.85) | 994 (77.96) |  |
| >=1.030 | 76 (4.35) | 9 (1.90) | 67 (5.25) |  |
| PRO, n(%) |  |  |  | **<.001** |
| Negative | 1165 (66.53) | 412 (86.74) | 753 (59.01) |  |
| Positive | 586 (33.47) | 63 (13.26) | 523 (40.99) |  |
| URO, n(%) |  |  |  | 0.164 |
| Negative | 1719 (98.23) | 470 (98.95) | 1249 (97.96) |  |
| Positive | 31 (1.77) | 5 (1.05) | 26 (2.04) |  |
| NIT, n(%) |  |  |  | **<.001** |
| Negative | 1610 (92.11) | 469 (99.15) | 1141 (89.49) |  |
| Positive | 138 (7.89) | 4 (0.85) | 134 (10.51) |  |

M: Median, Q₁: 1st Quartile, Q₃: 3st Quartile

Scr, serum creatinine; ALT, alanine aminotransferase; TP, serum total protein; TBA, total bile acid; GGT, γ-glutamyl transpeptidase; TC, total cholesterol; TG, triglyceride; HDL-C, high density lipoprotein cholesterol; LDL-C, low density lipoprotein cholesterol; UA, uric acid; WBC, white blood cell; NEUT#, neutrophil count; LYMPH#, lymphocyte count; MONO#, monocyte count; RBC, red blood cell; Hb, hemoglobin; PLT, platelet; KET, urine ketone bodies; SG, urine specific gravity; PRO, urine protein; URO, urobilinogen; NIT, urine nitrite.

**Supplementary Table S2. Univariate and multivariate analysis for analyzing the risk factors of CaOx stones before matching of gender and age**

| Variables | Univariate Unadjusted | | | | |  | Multivariate Adjusted | | | | |
| --- | --- | --- | --- | --- | --- | --- | --- | --- | --- | --- | --- |
|  | β | S.E | Z | *P* | OR (95%CI) |  | β | S.E | Z | *P* | OR (95%CI) |
| Gender |  |  |  |  |  |  |  |  |  |  |  |
| Male |  |  |  |  | 1.00 (Reference) |  |  |  |  |  |  |
| Female | -1.62 | 0.10 | -16.41 | **<.001** | 0.20 (0.16 ~ 0.24) |  |  |  |  |  |  |
| Age | 0.10 | 0.01 | 19.07 | **<.001** | 1.10 (1.09 ~ 1.11) |  | 0.11 | 0.01 | 8.49 | **<.001** | 1.11 (1.09 ~ 1.14) |
| KET |  |  |  |  |  |  |  |  |  |  |  |
| Negative |  |  |  |  | 1.00 (Reference) |  |  |  |  |  | 1.00 (Reference) |
| Trace | 2.23 | 1.02 | 2.19 | **0.029** | 9.32 (1.26 ~ 68.93) |  | 3.22 | 1.25 | 2.58 | **0.010** | 25.09 (2.18 ~ 289.14) |
| Positive | -0.29 | 0.61 | -0.48 | 0.633 | 0.75 (0.22 ~ 2.49) |  | -2.07 | 4.18 | -0.50 | 0.619 | 0.13 (0.00 ~ 450.57) |
| SG |  |  |  |  |  |  |  |  |  |  |  |
| <=1.005 |  |  |  |  | 1.00 (Reference) |  |  |  |  |  | 1.00 (Reference) |
| 1.005-1.030 | 0.19 | 0.14 | 1.38 | 0.167 | 1.21 (0.92 ~ 1.58) |  | -0.34 | 0.27 | -1.27 | 0.203 | 0.71 (0.42 ~ 1.20) |
| >=1.030 | 1.21 | 0.38 | 3.21 | **0.001** | 3.34 (1.60 ~ 6.97) |  | 0.94 | 0.68 | 1.38 | 0.168 | 2.57 (0.67 ~ 9.79) |
| PRO |  |  |  |  |  |  |  |  |  |  |  |
| Negative |  |  |  |  | 1.00 (Reference) |  |  |  |  |  | 1.00 (Reference) |
| Positive | 1.51 | 0.15 | 10.31 | **<.001** | 4.54 (3.41 ~ 6.06) |  | 0.94 | 0.30 | 3.11 | **0.002** | 2.57 (1.42 ~ 4.65) |
| URO |  |  |  |  |  |  |  |  |  |  |  |
| Negative |  |  |  |  | 1.00 (Reference) |  |  |  |  |  |  |
| Positive | 0.67 | 0.49 | 1.37 | 0.172 | 1.96 (0.75 ~ 5.13) |  |  |  |  |  |  |
| NIT |  |  |  |  |  |  |  |  |  |  |  |
| Negative |  |  |  |  | 1.00 (Reference) |  |  |  |  |  | 1.00 (Reference) |
| Positive | 2.62 | 0.51 | 5.14 | **<.001** | 13.77 (5.06 ~ 37.44) |  | 1.67 | 0.70 | 2.38 | **0.017** | 5.30 (1.35 ~ 20.88) |
| Scr (umol/L) | 0.06 | 0.00 | 18.28 | **<.001** | 1.06 (1.05 ~ 1.07) |  | 0.06 | 0.01 | 8.11 | **<.001** | 1.07 (1.05 ~ 1.08) |
| ALT (U/L) | 0.01 | 0.00 | 3.99 | **<.001** | 1.01 (1.01 ~ 1.02) |  | 0.03 | 0.01 | 3.90 | **<.001** | 1.03 (1.02 ~ 1.05) |
| TP (g/L) | -0.14 | 0.01 | -13.98 | **<.001** | 0.87 (0.85 ~ 0.89) |  | -0.07 | 0.02 | -2.98 | **0.003** | 0.94 (0.90 ~ 0.98) |
| TBA (umol/L) | 0.01 | 0.01 | 1.06 | 0.291 | 1.01 (0.99 ~ 1.02) |  |  |  |  |  |  |
| GGT (U/L) | 0.01 | 0.00 | 3.29 | **0.001** | 1.01 (1.01 ~ 1.01) |  | -0.02 | 0.01 | -3.47 | **<.001** | 0.98 (0.97 ~ 0.99) |
| TG (mmol/L) | 0.57 | 0.06 | 10.15 | **<.001** | 1.76 (1.58 ~ 1.96) |  | 0.37 | 0.12 | 3.07 | **0.002** | 1.44 (1.14 ~ 1.82) |
| HDL-C (mmol/L) | -2.67 | 0.18 | -14.54 | **<.001** | 0.07 (0.05 ~ 0.10) |  | -1.06 | 0.37 | -2.89 | **0.004** | 0.35 (0.17 ~ 0.71) |
| LDL-C (mmol/L) | -0.33 | 0.06 | -5.21 | **<.001** | 0.72 (0.63 ~ 0.81) |  | -0.43 | 0.15 | -2.78 | **0.005** | 0.65 (0.48 ~ 0.88) |
| UA (umol/L) | 0.01 | 0.00 | 12.20 | **<.001** | 1.01 (1.01 ~ 1.01) |  |  |  |  |  |  |
| WBC (10^9/L) | 0.34 | 0.03 | 11.89 | **<.001** | 1.40 (1.33 ~ 1.48) |  | 0.36 | 0.19 | 1.85 | 0.064 | 1.43 (0.98 ~ 2.08) |
| NEUT# (10^9/L) | 0.52 | 0.04 | 13.44 | **<.001** | 1.68 (1.55 ~ 1.81) |  | 0.48 | 0.21 | 2.32 | **0.020** | 1.61 (1.08 ~ 2.42) |
| LYMPH# (10^9/L) | -0.06 | 0.07 | -0.87 | 0.386 | 0.94 (0.83 ~ 1.08) |  |  |  |  |  |  |
| MONO# (10^9/L) | -0.91 | 0.27 | -3.33 | **<.001** | 0.40 (0.24 ~ 0.69) |  | -8.94 | 1.15 | -7.76 | **<.001** | 0.00 (0.00 ~ 0.00) |
| RBC (10^12/L) | 0.77 | 0.08 | 9.74 | **<.001** | 2.16 (1.85 ~ 2.52) |  | 0.99 | 0.21 | 4.75 | **<.001** | 2.69 (1.79 ~ 4.05) |
| Hb (g/L) | 0.00 | 0.00 | 1.13 | 0.257 | 1.00 (1.00 ~ 1.01) |  |  |  |  |  |  |
| PLT (10^9/L) | -0.00 | 0.00 | -1.79 | 0.074 | 1.00 (1.00 ~ 1.00) |  |  |  |  |  |  |
| Urine pH | 0.74 | 0.10 | 7.46 | **<.001** | 2.10 (1.73 ~ 2.55) |  | 0.91 | 0.18 | 5.12 | **<.001** | 2.49 (1.75 ~ 3.52) |
| DBIL/Alb (100 times) | -0.04 | 0.02 | -1.66 | 0.097 | 0.97 (0.93 ~ 1.01) |  |  |  |  |  |  |
| UCB/Alb (100 times) | 0.10 | 0.01 | 15.23 | **<.001** | 1.11 (1.10 ~ 1.13) |  | 0.07 | 0.02 | 4.29 | **<.001** | 1.07 (1.04 ~ 1.11) |
| OR: Odds Ratio, CI: Confidence Interval | | | | | | | | | | | |

Scr, serum creatinine; ALT, alanine aminotransferase; TP, serum total protein; TBA, total bile acid; GGT, γ-glutamyl transpeptidase; TG, triglyceride; HDL-C, high density lipoprotein cholesterol; LDL-C, low density lipoprotein cholesterol; UA, uric acid; WBC, white blood cell; NEUT#, neutrophil count; LYMPH#, lymphocyte count; MONO#, monocyte count; RBC, red blood cell; Hb, hemoglobin; PLT, platelet; KET, urine ketone bodies; SG, urine specific gravity; PRO, urine protein; URO, urobilinogen; NIT, urine nitrite.

**Supplementary Table S3. Gender, Age and laboratory information by the status of CaOx stones after matching of gender and age**

| Variables | Total  (n = 854) | Control  (n = 427) | CaOx-urolithiasis  (n = 427) | *P* |
| --- | --- | --- | --- | --- |
|  |  |  |  |  |
| Scr (umol/L), M (Q₁, Q₃) | 81.45 (68.60, 95.82) | 76.60 (66.70, 89.70) | 86.40 (72.10, 105.30) | **<.001** |
| ALT (U/L), M (Q₁, Q₃) | 19.00 (14.00, 28.00) | 18.40 (13.50, 27.95) | 19.00 (14.00, 28.00) | 0.182 |
| TP (g/L), M (Q₁, Q₃) | 75.30 (72.00, 78.10) | 76.40 (73.85, 78.95) | 73.60 (69.55, 76.85) | **<.001** |
| TBA (umol/L), M (Q₁, Q₃) | 4.38 (3.10, 6.70) | 4.80 (3.80, 6.55) | 3.78 (2.12, 6.97) | **<.001** |
| GGT (U/L), M (Q₁, Q₃) | 20.00 (13.93, 30.08) | 21.20 (15.30, 31.85) | 18.00 (12.00, 28.00) | **<.001** |
| TC (mmol/L), M (Q₁, Q₃) | 5.22 (4.66, 5.87) | 5.18 (4.62, 5.78) | 5.36 (4.72, 5.93) | 0.058 |
| TG (mmol/L), M (Q₁, Q₃) | 1.32 (0.92, 2.03) | 1.20 (0.90, 1.83) | 1.54 (0.98, 2.49) | **<.001** |
| LDL-C (mmol/L), Mean ± SD | 3.35 ± 0.80 | 3.50 ± 0.74 | 3.10 ± 0.84 | **<.001** |
| HDL-C (mmol/L), M (Q₁, Q₃) | 1.39 (1.17, 1.62) | 1.45 (1.24, 1.67) | 1.26 (1.05, 1.50) | **<.001** |
| UA (umol/L), M (Q₁, Q₃) | 371.00 (318.00, 447.95) | 361.40 (306.35, 436.05) | 381.00 (329.00, 461.00) | **0.021** |
| WBC (10^9/L), M (Q₁, Q₃) | 6.40 (5.41, 7.60) | 6.00 (5.10, 6.90) | 6.91 (5.79, 8.19) | **<.001** |
| NEUT# (10^9/L), M (Q₁, Q₃) | 3.60 (2.90, 4.70) | 3.20 (2.70, 3.90) | 4.40 (3.30, 5.70) | **<.001** |
| LYMPH# (10^9/L), M (Q₁, Q₃) | 2.00 (1.70, 2.50) | 2.10 (1.70, 2.50) | 2.00 (1.60, 2.50) | **0.039** |
| MONO# (10^9/L), M (Q₁, Q₃) | 0.40 (0.30, 0.50) | 0.40 (0.40, 0.50) | 0.40 (0.30, 0.50) | **<.001** |
| RBC (10^12/L), M (Q₁, Q₃) | 4.79 (4.42, 5.16) | 4.71 (4.39, 5.03) | 4.92 (4.50, 5.33) | **<.001** |
| Hb (g/L), M (Q₁, Q₃) | 138.00 (128.00, 151.00) | 141.00 (132.00, 152.00) | 136.00 (123.00, 149.00) | **<.001** |
| PLT (10^9/L), M (Q₁, Q₃) | 239.00 (206.00, 281.00) | 236.00 (206.25, 269.75) | 241.00 (206.50, 291.00) | 0.080 |
| Urine pH, M (Q₁, Q₃) | 6.00 (6.00, 6.50) | 6.00 (5.50, 6.50) | 6.00 (6.00, 6.50) | **0.004** |
| KET, n(%) |  |  |  | 0.090 |
| Negative | 623 (97.34) | 225 (99.12) | 398 (96.37) |  |
| Trace | 12 (1.88) | 1 (0.44) | 11 (2.66) |  |
| Positive | 5 (0.78) | 1 (0.44) | 4 (0.97) |  |
| SG, n(%) |  |  |  | 0.264 |
| <=1.005 | 133 (20.65) | 45 (19.48) | 88 (21.31) |  |
| 1.005-1.030 | 481 (74.69) | 179 (77.49) | 302 (73.12) |  |
| >=1.030 | 30 (4.66) | 7 (3.03) | 23 (5.57) |  |
| PRO, n(%) |  |  |  | **<.001** |
| Negative | 456 (70.81) | 203 (88.26) | 253 (61.11) |  |
| Positive | 188 (29.19) | 27 (11.74) | 161 (38.89) |  |
| URO, n(%) |  |  |  | 0.090 |
| Negative | 631 (98.13) | 229 (99.57) | 402 (97.34) |  |
| Positive | 12 (1.87) | 1 (0.43) | 11 (2.66) |  |
| NIT, n(%) |  |  |  | **<.001** |
| Negative | 593 (92.22) | 229 (99.57) | 364 (88.14) |  |
| Positive | 50 (7.78) | 1 (0.43) | 49 (11.86) |  |

M: Median, Q₁: 1st Quartile, Q₃: 3st Quartile, SD: Standard deviation

Scr, serum creatinine; ALT, alanine aminotransferase; TP, serum total protein; TBA, total bile acid; GGT, γ-glutamyl transpeptidase; TC, total cholesterol; TG, triglyceride; HDL-C, high density lipoprotein cholesterol; LDL-C, low density lipoprotein cholesterol; UA, uric acid; WBC, white blood cell; NEUT#, neutrophil count; LYMPH#, lymphocyte count; MONO#, monocyte count; RBC, red blood cell; Hb, hemoglobin; PLT, platelet; KET, urine ketone bodies; SG, urine specific gravity; PRO, urine protein; URO, urobilinogen; NIT, urine nitrite.

**Supplementary Table S4. Table of Primers sequences**

| Gene/Specie |  | Oligonucleotides |
| --- | --- | --- |
| *Slc26a6* | forward | 5-GCCCAGCGTCAGAGGAAA-3' |
|  | reverse | 5-ACCAGCATAGATGCGACCATA-3' |
| *β-tubulin* | forward | 5-ATCAGCAAGATCCGAGAAGA-3' |
|  | reverse | 5-GAGGGTGGCATTGTAGGG-3' |
| *GAPDH* | forward | 5-CAAGGCTGTGGGCAAGGT-3' |
|  | reverse | 5-CGAAGGTGGAAGAGTGGGT-3' |
| *Pristin* | forward | 5-GACGGCAAAGGTGGTGGATA-3' |
|  | reverse | 5-AGGAGACAGAATCCGCAAGC-3' |
| *RpL32* | forward | 5-AGCATACAGGCCCAAGATCG-3' |
|  | reverse | 5-GTTGTCGATACCCTTGGGCT-3' |
| β-GD (Unigene2520899)  *Bacteroides faecichinchillae* | forward | 5-TGCGTGAATATGTCTCTGGCAAC-3' |
|  | reverse | 5-TAACGGGAGTCGTATGCGTAAGT-3' |
| β-GD (Unigene160707)  *Bacteroides fragilis* | forward | 5-GCGAAGGCATCATTTCTTTCTATTA-3' |
|  | reverse | 5-TCTCGCCATCTTTCAGGTATTT-3' |
| β-GD (Unigene2857124)  *Bacteroides thetaiotaomicron* | forward | 5-TCATAAGGGTTCGGAAATGCGTG-3' |
|  | reverse | 5-CGTCCCATGAAAGAAAGCATGGA-3' |
| β-GD (Unigene2510810)  *Muribaculaceae bacterium Isolate-042 (Harlan)* | forward | 5-ACATCAGGAAGACAGGTAGTGGG-3' |
|  | reverse | 5-TAGGGGCAGTGAAGTCGATTGAT-3' |
| β-GD (Unigene21381)  *bacterium J10(2018)* | forward | 5-GAATCATACCCATACTTCCTTGAG-3' |
|  | reverse | 5-GTTCTCGACCTGCACCATTATTA-3' |
| β-GD (Unigene161415)  *Bacteroidales bacterium* | forward | 5-ATATTCTGGAGTCTGGGCAACGA-3' |
|  | reverse | 5-GTGCCTGTTCATATTGCACCGTA-3' |
| β-GD (Unigene124978)  *Bacteroides uniformis* | forward | 5-AAAACAATATGACCCCTCACGCC-3' |
|  | reverse | 5-CCTATGACATTTGCACGCTTTGC-3' |
| β-GD (Unigene148681)  *Bacteroides oleiciplenus* | forward | 5-GCGTACCGGGAAGTTTGTCTATG-3' |
|  | reverse | 5-ATCGTATAGTCCACGGGAACCTG-3' |
| β-GD (Unigene114408)  *Muribaculaceae bacterium Isolate-104 (HZI)* | forward | 5-TCAGGTGGAAATCAAGGAAACGC-3' |
|  | reverse | 5-GCATGACCGAAAACAGGAGATGT-3' |
| β-GD (Unigene114591)  *Muribaculaceae bacterium Isolate-037 (Harlan)* | forward | 5-CATGAACTCCACCTATCCCGACT-3' |
|  | reverse | 5-CCGTATATTTGGAAGCCTCGCTG-3' |
| *BilR* (*Unigene1600136*)  *Eubacterium plexicaudatum* | forward | 5-GGCCGTGATAGAGATGCTGGATA-3' |
|  | reverse | 5-TGGTCAGCAAAATCCTTCTGGATG-3' |
| *BilR* (*Unigene1127320*) | forward | 5-GCAGGAAAATCCTCATTACGGCA-3' |
|  | reverse | 5-AAAACTGGTAACACCGGCCTTTT-3' |
| *BilR* (*Unigene11122212*) | forward | 5-CAGGTATTGCTGGATTGGAAGCA-3' |
|  | reverse | 5-TGGAGGGATAGCTGCAAGATGAA-3' |
| *BilR* (*Unigene1316070*) | forward | 5-CAGCAACTGGAGGAGATCAAA-3' |
|  | reverse | 5-TTGAGCATTTCGGAGCAGAG-3' |
| *BilR* (*Unigene1067885*) | forward | 5-CACCTGCCTTAACTGCAACA-3' |
|  | reverse | 5-GCGTTGAGTACACAGGAGATATAC-3' |
| *16S rDNA* | 341F | CCTACGGGNGGCWGCAG |
|  | 806R | GGACTACHVGGGTATCTAAT |
